# SOAR elucidates biological insights and empowers drug discovery through spatial transcriptomics

**DOI:** 10.1126/sciadv.adt7450

**Published:** 2025-06-11

**Authors:** Yiming Li, Yanyi Ding, Saya Dennis, Meghan R. Hutch, Jiaqi Zhou, Yadi Zhou, Yawei Li, Maalavika Pillai, Sanaz Ghotbaldini, Mario Alberto Garcia, Mia S. Broad, Chengsheng Mao, Parambir S. Dulai, Feixiong Cheng, Zexian Zeng, Yuan Luo

**Affiliations:** ^1^ Department of Preventive Medicine, Northwestern University, Chicago, IL 60611, USA.; ^2^ Data Science Institute, University of Chicago, Chicago, IL 60637, USA.; ^3^ Genomic Medicine Institute, Lerner Research Institute, Cleveland Clinic, Cleveland, OH 44195, USA.; ^4^ Ken and Ruth Davee Department of Neurology, Northwestern University Feinberg School of Medicine, Chicago, IL 60610, USA.; ^5^ Department of Medicine, Division of Gastroenterology and Hepatology, Northwestern University, Chicago, IL 60611, USA.; ^6^ Center for Human Immunobiology, Northwestern University, Chicago, IL 60611, USA.; ^7^ Department of Molecular Medicine, Cleveland Clinic Lerner College of Medicine, Case Western Reserve University, Cleveland, OH 44106, USA.; ^8^ Case Comprehensive Cancer Center, Case Western Reserve University, Cleveland, OH 44106, USA.; ^9^ Center for Quantitative Biology, Academy for Advanced Interdisciplinary Studies, Peking University, Beijing 100871, China.; ^10^ Peking-Tsinghua Center for Life Sciences, Academy for Advanced Interdisciplinary Studies, Peking University, Beijing 100871, China.; ^11^ Northwestern University Clinical and Translational Sciences Institute, Northwestern University, Chicago, IL 60611, USA.; ^12^ Center for Collaborative AI in Healthcare, Institute for AI in Medicine, Northwestern University, Chicago, IL 60611, USA.

## Abstract

Spatial transcriptomics enables multiplex profiling of gene cellular expression and location within the tissue context. Although large volumes of spatial transcriptomics data have been generated, the lack of systematic curation and analysis limits biological discovery. We present Spatial transcriptOmics Analysis Resource (SOAR), a comprehensive spatial transcriptomics platform with 3461 uniformly processed samples across 13 species, 42 tissue types, and 19 different spatial transcriptomics technologies. Using SOAR, we found that *CXCL16*/*SPP1* macrophage polarity characterizes the coordination of immune cell polarity in the tumor microenvironment. SOAR’s integrative approach toward drug discovery revealed sirolimus and trichostatin A as potential anticancer agents targeting the phosphatidylinositol 3-kinase/Akt/mammalian target of rapamycin growth and proliferation pathway and identified Janus kinase/signal transducers and activators of transcription inhibitors for ulcerative colitis treatment. SOAR’s results demonstrate its broad application to data generated from diverse spatial technologies and pathological conditions. SOAR will support future benchmarking studies and method development, facilitating discoveries in molecular functions, disease mechanisms, and potential therapeutic targets.

## Teaser

SOAR is a comprehensive spatial transcriptomics platform for uncovering biological insights and potential therapeutic targets.

## INTRODUCTION

Spatially resolved transcriptomics preserves the spatial organization of cells, allowing researchers to study molecular functions and disease pathology within their native morphological context (*1*–*5*). Different spatial transcriptomics technologies have been developed, leading to discoveries in cancer, neuroscience, and developmental biology (*3*, *6*–*9*). These advancements have shed light on complex biological insights and paved the way for insights into human traits and disorders.

Despite technological advancements, systematically accessing and analyzing spatial transcriptomics data remains a challenge. Multiple data resources have been developed to address this need (*10*–*19*), each with unique strengths and limitations. To bridge existing gaps, we present Spatial transcriptOmics Analysis Resource (SOAR; https://soar.fsm.northwestern.edu/), a publicly available spatial transcriptomics platform with an extensive collection of spatial transcriptomics datasets, diverse analytical tools, and interactive visualization features. A systematic comparison between SOAR and existing resources is provided in table S1.

Early resources such as the Museum of Spatial Transcriptomics (*10*) provide literature annotations rather than processed datasets, while data atlases like spatialLIBD (*11*), STAR-FINDer (*12*), the spatiotemporal transcriptome atlas of the Amyotrophic lateral sclerosis spinal cord (ALS-ST) (*13*), and SORC (*14*) are focused on specific tissue types or diseases (brain, intestine, spinal cord, and cancer, respectively), limiting their broader applicability. In response, databases such as SpatialDB (*15*), STOmicsDB (*16*), SPASCER (*17*), SODB (*18*), and Aquila (*19*) were developed, each varying in size and functionality. In terms of volume, SpatialDB contains 305 spatial transcriptomics samples, STOmicsDB hosts 2438 samples with spatial coordinates, SPASCER has 1082 samples, SODB hosts 1701 samples, and Aquila consists of 1397 samples. SOAR surpasses these platforms by hosting 3461 spatial transcriptomics samples with coordinates data, making it the most extensive resource available (table S1).

While many existing platforms provide useful functions such as spatial visualization, clustering, cell typing, and spatial variability analysis, they often lack key analytical tools like cell-cell interaction (CCI), mega-analysis, and drug discovery. Firstly, spatial CCI analysis is a major strength of spatial transcriptomics, but only SPASCER (*17*) and Aquila (*19*) support neighborhood-based CCI analysis. SOAR expands on this by providing both neighborhood-based and distance-based CCI analyses. Secondly, mega-analysis across datasets, which is essential for uncovering broader biological insights, is currently limited to SPASCER (*17*) and ALS-ST (*13*). SOAR supports mega-analysis, allowing researchers to compare and integrate findings across studies. Last, existing platforms do not provide drug discovery tools, leaving a gap in translating spatial transcriptomics data into therapeutic applications. SOAR addresses this need with a drug discovery module, further enhancing its utility for biomedical research.

**Fig. 1.**
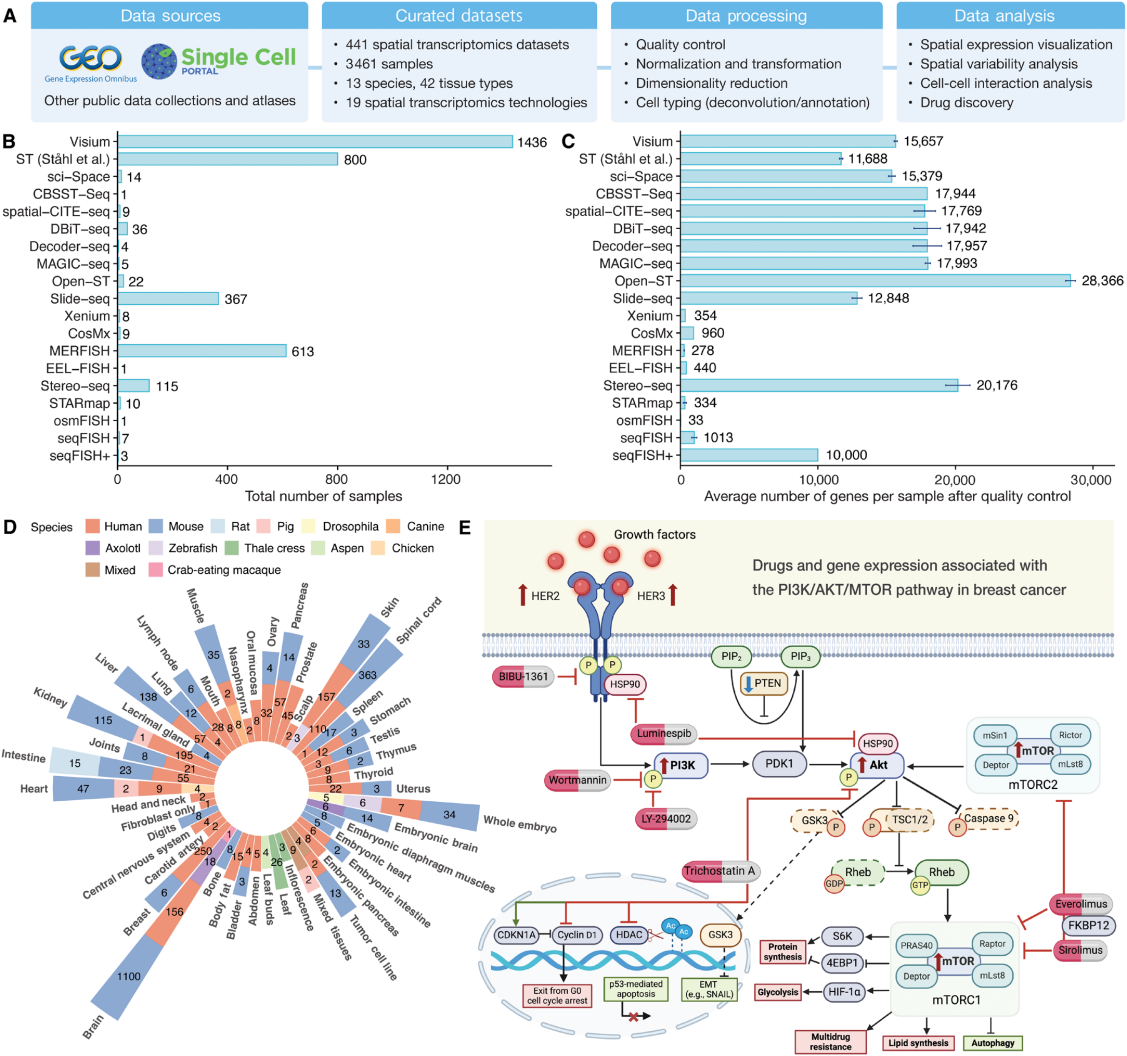
Overview of SOAR and drugs targeting genes in the PI3K/Akt/mTOR pathway in breast cancer. (**A**) Datasets hosted on SOAR were curated from public domains and processed using a standardized workflow. SOAR also provides interactive interfaces for users to visualize spatial gene expressions, evaluate the spatial variability of genes, assess CCIs, and conduct drug discovery. (**B** and **C**) Statistics of data from different spatial transcriptomics technologies in SOAR. The 95% confidence intervals for the means are plotted as error bars. (**D**) Number of samples of each species and tissue type combination. SOAR contains 3461 spatial transcriptomics samples from 13 different species across 42 tissue types. (**E**) The drug discovery function of SOAR identifies various compounds that target the PI3K/Akt/mTOR growth and proliferation pathway and show strong suppression effects toward DEGs in malignant cells. Up-regulated (red upward arrow) and down-regulated (blue downward arrow) PI3K/Akt/mTOR genes are shown.

Overall, these limitations in scale, scope, and analysis functionality restrict comprehensive exploration of spatial transcriptomics data and hinder the broader applications in disease modeling, pathway analysis, and therapeutic development. SOAR addresses these challenges by providing a unified platform for exploring spatial gene expressions and performing spatial variability, CCIs, and drug discovery analysis (Fig. 1A). To demonstrate SOAR’s capabilities, we present multiple case studies across cancer and noncancer disorders. Using SOAR’s spatial variability and CCI functions, we found that *CXCL16*/*SPP1* macrophage polarity characterizes the coordination of immune cell polarity in the tumor microenvironment (TME). Through SOAR’s drug discovery module, we identified compounds that inhibit the phosphatidylinositol 3-kinase (PI3K)/Akt/mammalian target of rapamycin (mTOR) growth and proliferation pathway in breast cancer and compounds that suppress the Janus kinase (JAK)/signal transducers and activators of transcription (STAT) proinflammatory signaling pathway in ulcerative colitis (UC). By integrating large-scale spatial transcriptomics data with advanced analytical tools, SOAR serves as a comprehensive resource that enables the discovery of biological insights and potential therapeutic targets.

## RESULTS

### Platform data summary

SOAR is a novel spatial transcriptomics platform to offer deep drug discovery insights across a variety of disorders. SOAR hosts the largest collection of spatial transcriptomics data with spatial coordinates data, which can facilitate future mega-analysis, benchmarking studies, and method development (table S1). Currently, SOAR hosts 3461 samples across 19 different spatial transcriptomics technologies (Fig. 1, B and C, and figs. S1 and S2A), 13 species, and 42 tissue types (Fig. 1D and fig. S2B) from 441 datasets (table S2). The datasets were reviewed, annotated, and preprocessed through a standardized workflow (Materials and Methods). In SOAR’s data browser, users can browse the curated datasets to pinpoint samples of interest using metadata-based filters and visualize spatial expressions interactively. SOAR also provides interactive functions, including visualization of spatial gene expression and clustering results, exploration of spatial variability, analysis of neighborhood-based and distance-based CCIs, discovery of potential drugs, and mega-analyses across datasets (Fig. 1A and table S1). The processed data, including normalized expressions, images, coordinates, and phenotypic data, are publicly available for download. In summary, SOAR is a comprehensive resource that could facilitate the understanding of gene cellular expression patterns and disease pathology in the tissue context as well as therapeutic discovery.

### Spatial gene expression patterns in the TME

**Fig. 2.**
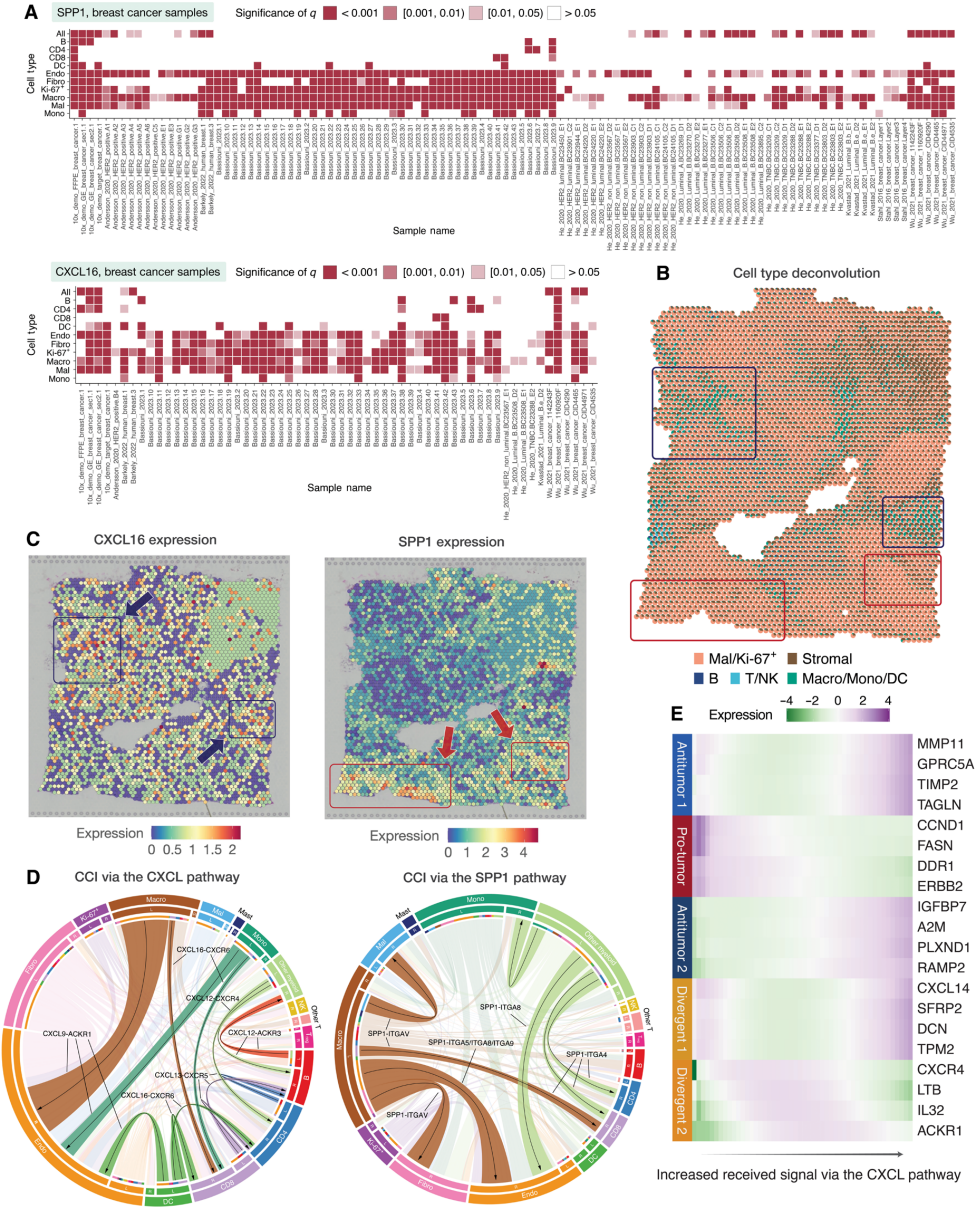
Explore spatial variability, CCI, and DEGs in cancer spatial transcriptomics data in SOAR. (**A**) Spatial variability of *CXCL16* and *SPP1* in breast cancer samples. The color indicates the significance level of spatial variability of the gene in a certain cell type, sample combination. *P* values are adjusted using the false discovery approach. (**B**) Deconvolution results of a breast cancer sample. The red boxes indicate regions with high proportions of malignant and Ki-67^+^ cells, and the blue boxes indicate areas of high immune cell proportions. (**C**) Spatial gene expressions of *CXCL16* and *SPP1* in a breast cancer sample. The arrows indicate distinct regions of elevated expression of the genes. (**D**) Pan-cancer short-distance (500 μm) CCIs among different cell types through the CXCL and SPP1 pathways. The top ligand-receptor pairs are highlighted. (**E**) Heatmap of selected genes differentially expressed with respect to the amount of received signal through the CXCL pathway in a breast cancer sample. Abbreviations: DC, dendritic cells; Endo, endothelial cells; Fibro, fibroblasts; Macro, macrophages; Mal, malignant cells; Mono, monocytes; NK, natural killer cells; T_reg_, regulatory T cells; *q*, false discovery rate–adjusted *P* value.

The spatial patterns of gene expression inform us about the migration of cells, their responses to different tissue environments, and CCIs (*1*, *20*). SOAR allows users to evaluate the statistical significance of a gene’s spatial variability in different tissue samples and cell types. Chemokine genes, such as *CXCL16*, are known to be associated with immune activation and the prognosis of cancer (*21*, *22*), and their expression levels were found to be spatially variable in cancer samples (*20*, *23*). *SPP1* (secreted phosphoprotein 1) has been shown to be overexpressed in different types of tumors (*24*, *25*) and promote tumor progression and metastasis (*26*, *27*). Therefore, as a case study, we visualized the spatial variability of *CXCL16* and *SPP1* in our curated breast cancer samples using SOAR (Fig. 2A). Results from SOAR suggested significant spatial variation of both genes in most of the breast cancer samples (Fig. 2A). Specifically, these two genes have more frequent spatial variability (adjusted *P* value <0.05 ) in macrophages compared to other cell types, indicating their cell type specificity in the tumor immune microenvironment (Fig. 2A).

In addition, our analyses revealed that *CXCL16* and *SPP1* colocalize with different cell types (Fig. 2, B and C). *CXCL16* was highly expressed in areas enriched with T and natural killer (NK) cells (Kendall’s rank correlation coefficient = 0.06, *P* value = 3.27 × 10^−6^) (Fig. 2, B and C). These findings are consistent with previous studies showing that T and NK cells induce *CXCL16* expression, which promotes inflammation and recruits immune cells to sites of malignancy (*28*, *29*). *CXCL16* plays a key role in amplifying local immune responses, with its high expression reflecting an active proinflammatory environment driven by immune cells that work to control tumor progression (*30*). Our analysis revealed that spatial areas with low *CXCL16* expression and aggregated malignant cells exhibited elevated levels of *SPP1* (Fig. 2, B and C). This spatial pattern aligns with previous findings that tumor progression is often accompanied by high *SPP1* expression in tumor-associated macrophages (TAMs), which support tumor growth and immunosuppression (*31*). In summary, SOAR was able to identify that *CXCL16* and *SPP1* are associated with proinflammatory and pro-tumor responses, respectively, aligning with previous findings (*20*, *21*, *24*, *32*). These results demonstrate that SOAR could reveal biologically relevant genes with colocalized expression patterns.

### CCI analysis reveals context-dependent gene functions

**Fig. 3.**
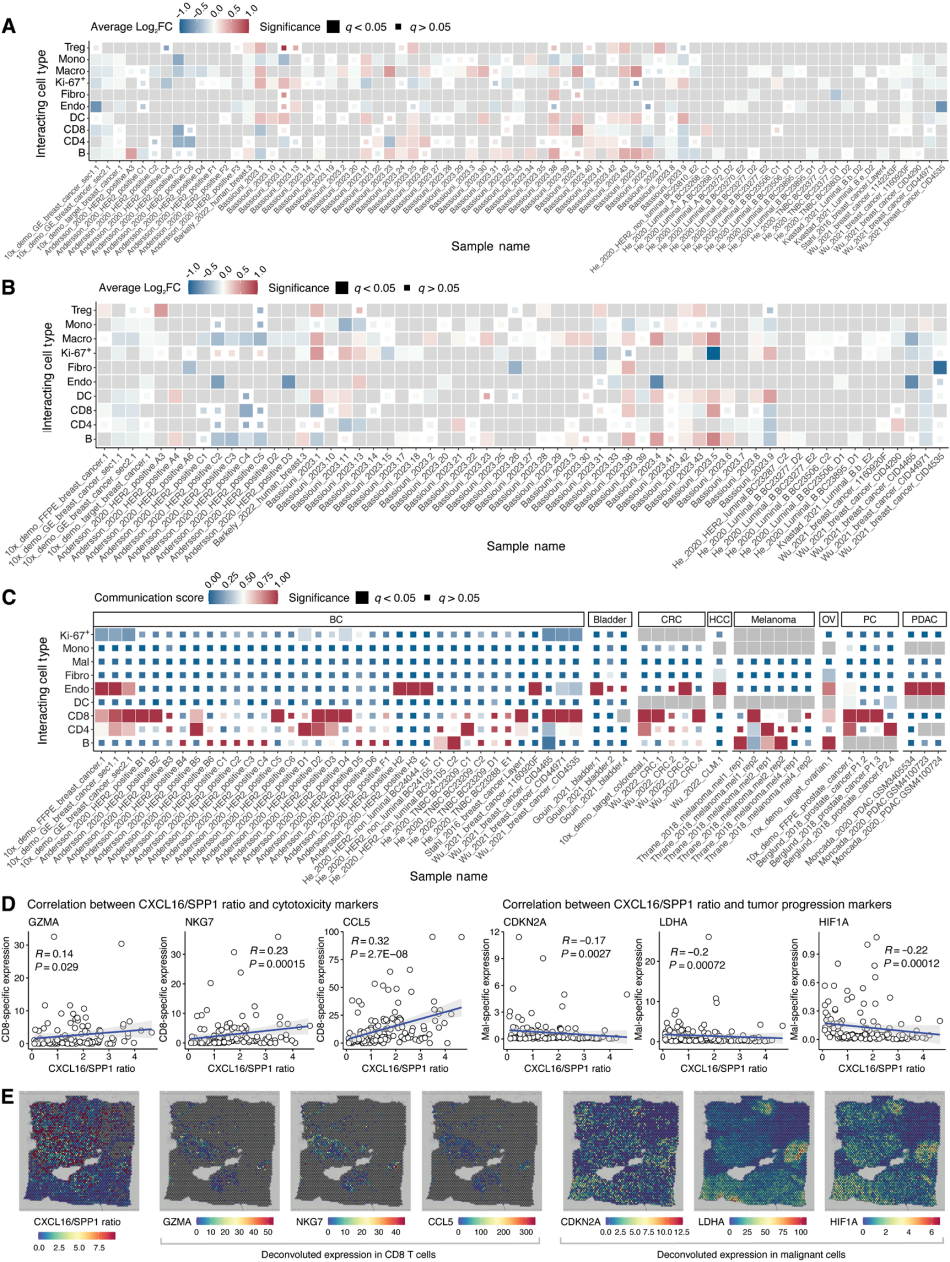
Assess neighborhood-based and distance-based CCIs in SOAR. Tumor-promoting complement (**A**) *C1QA* and (**B**) *C1QB* differentially express among malignant capture locations adjacent and nonadjacent to other cell types in breast cancer samples. Each tile in the heatmap is colored by the log-fold change in gene expression and sized according to its statistical significance after false discovery rate adjustment. A gray tile indicates that the cell type is unavailable in that sample. *P* values are adjusted using the false discovery rate approach. (**C**) Macrophages interact with T cells in cancer samples through the CXCL pathway. The tiles are colored by the communication scores, and gray color indicates that the cell type is not available in that sample. (**D** and **E**) *CXCL16*/*SPP1* ratio in macrophages correlates with the expression of cytotoxicity and tumor progression markers. *P* values are adjusted using the false discovery rate approach. Abbreviations: BC, breast cancer; BLCA, bladder cancer; CRC, colorectal cancer; HCC, liver cancer; Log_2_FC, log-fold changes; OV, ovarian cancer; PC, prostate cancer; PDAC, pancreatic ductal adenocarcinoma; RCC, renal cell carcinoma.

With SOAR, users could explore whether the expression of a specific gene is associated with the presence of nearby cell types (Fig. 3, A and B). Since cells could communicate with nonadjacent cells through the secretion of chemokines (*33*), SOAR also enables users to investigate more distant but biologically meaningful CCI signals (Figs. 2D and 3, C to E). As a proof of concept, we performed CCI analysis on cancer samples, focusing on chemokine signaling (mainly the *CXCL16-CXCR6* ligand-receptor pair) and the *SPP1* gene (mainly the *SPP1-ITGAV* interaction). We found that while the expression level of *CXCL16-CXCR6* axis is associated with inflammatory signaling pathways, *SPP1-ITGAV* is associated with tumor growth (Fig. 2D). In previous studies, *CXCR6*^+^ CD8 T cells were found to exhibit a stronger antitumor ability and demonstrated an enhanced response to PD-1 inhibitors (*34*). This finding was confirmed in SOAR, as our CCI analysis showed that *CXCL16*^+^ dendritic cells and myeloid cells interacted with *CXCR6*^+^ CD8 T cells, signifying a potential synergistic immune response against tumor (Fig. 2D). In addition, our analyses revealed that *CXCL13*^+^ T cells and *CXCR5*^+^ B/T cells increased immune cell recruitment and lymphocyte infiltration (Fig. 2D) (*35*, *36*). Furthermore, differential expression analysis showed that more signals from *CXCL16*-*CXCR6* and other chemokine-related CCIs are positively associated with the expressions tumor-suppressing genes, including *IGFBP7* (*37*), *A2M* (*38*), and *GPRCA5* (Fig. 2E) (*39*). Meanwhile, *CXCL16*-*CXCR6* expression is also negatively associated with tumor promoting genes, including *CCND1* (*40*), *ERBB2* (*41*), and *DDR1* (*42*) (Fig. 2E). In contrast, the CCIs involving *SPP1* occurred more frequently between macrophages and stromal cells rather than macrophages and other immune cells (Fig. 2D). For example, *SPP1*^+^ macrophages strongly interacted with *ITGAV*^+^ endothelial cells, fibroblasts, and malignant cells (Fig. 2D). *SPP1*’s involvement in the integrin pathway has been reported to be involved in metastatic seeding (*43*) and activation of the Akt pathway (*25*). The silencing of *ITGAV* can, in turn, inhibit tumor growth (*43*). In addition, *SPP1*^+^ macrophages also interacted with *CD44*^+^ mast cells (Fig. 2D). Previous studies showed that the *SPP1*-*CD44* interaction could hinder mast cells’ ability to activate T cells in the TME (*44*, *45*). To further investigate the impact of cell type adjacency on gene expression, we performed neighborhood-based CCI analysis. Our analyses showed that when tumor cells are in proximity with macrophages, the complement genes *C1QA*/*C1QB* have higher expression (Fig. 3, A and B). This result suggested positive interactions between malignant cells and TAMs evading immune surveillance as well as leading to cancer cell adhesion and migration proliferation. In sum, SOAR was able to identify that CCIs involving *CXCL16*-*CXCR6* may lead to inflammatory responses, whereas those related to *SPP1*-*ITGAV* may result in an immune-suppressive environment.

To further investigate whether *CXCL16*/*SPP1* macrophage polarity is associated with tumor control and progression, we analyzed the correlations between the macrophage *CXCL16*/*SPP1* ratio and the expression of cytotoxicity markers and tumor progression markers. SOAR’s results showed that the *CXCL16*/*SPP1* ratio was positively correlated with the presence of cytotoxic CD8 T cells, identified by the markers *GZMA* (granzyme A) (*46*, *47*) and *LAG3* (lymphocyte activation gene 3) (*48*). In addition, the macrophage *CXCL16*/*SPP1* ratio is negatively correlated with the expression of genes responsible for hypoxia-induced glycolysis in tumors, such as *LDHA* (lactate dehydrogenase A) and *HIF1A* (hypoxia-inducible factor 1 subunit alpha) (Figs. 3D and fig. S3) (*49*, *50*). As an example, these trends can be observed in the spatial visualizations of *CXCL16*/*SPP1* ratio as well as cytotoxicity and tumor progression markers expression levels in 10x_demo_GE_breast_cancer_sec1.1 (Fig. 3E). Together, these results from SOAR suggest that macrophage polarity on the *CXCL16*/*SPP1* axis could characterize the coordination of antitumor and pro-tumor pathways in the TME. This draws insightful parallels with recent findings that a higher *CXCL9*/*SPP1* ratio in macrophages is associated with increased tumor killing by immune cells and enhanced survival of patients with cancer (*32*). While increasing both chemokine ratios promotes a more immunologically active and potentially hostile TME against cancer cells.

### Drug discovery and repurposing analysis

#### Analysis overview and drug screening criteria

**Fig. 4.**
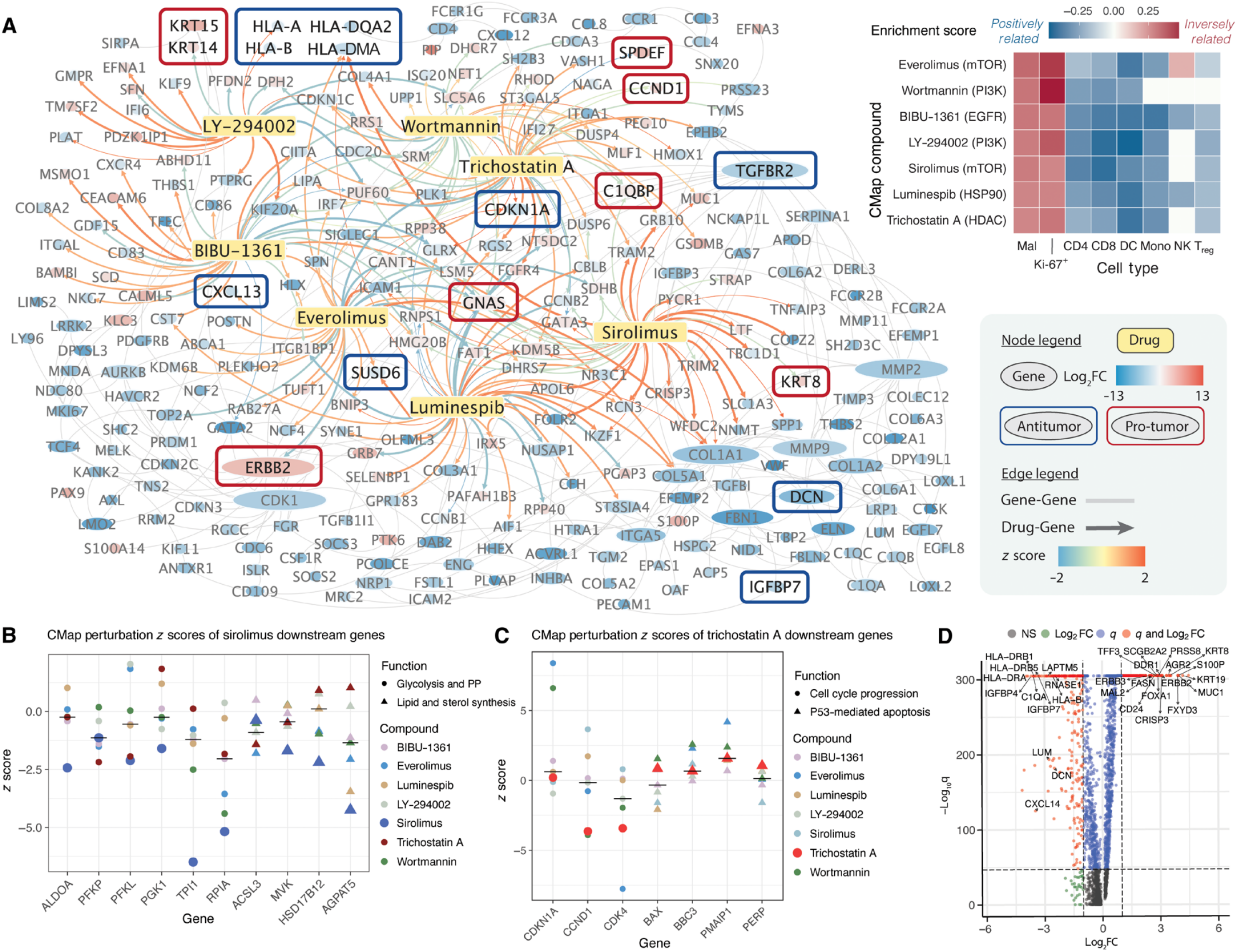
SOAR performs drug discovery in cancer spatial transcriptomics data. (**A**) PPI and drug perturbation network in a breast cancer sample. Gene nodes are colored by log-fold change in malignant cells and sized by total degree. Known antitumor and pro-tumor genes are boxed. Drug-gene edges are weighted by expression *z* scores from CMap. A heatmap visualizes ES of compounds targeting the PI3K/Akt/mTOR pathway across cell types, showing strong suppression of spatially variable and DEGs (red) in malignant and Ki-67^+^ cells, with neutral to promoting effects (blue) on those genes of immune cells. (**B**) Sirolimus can down-regulate energy metabolism and biosynthesis genes. Sirolimus down-regulates genes involved in glycolysis, pentose phosphate, lipid, and sterol synthesis, all mTORC1 targets. Perturbations of other case study drugs are shown as comparison. Black lines indicate median expression *z* scores. (**C**) TSA down-regulates cell cycle progression genes and up-regulate apoptosis genes. TSA reduces *CCND1* and *CDK4* expression (G_1_ cycle progression) and increases *CDKN1A* (cyclin-CDK inhibitor) and p53 downstream apoptosis genes. (**D**) Volcano plot highlights DEGs in malignant cells. Tumor-promoting and tumor-suppressing genes are labeled. Log-fold change threshold: 1; adjusted *P* value threshold: 10^−32^. Abbreviations: CMap, Connectivity Map; NS, non-significant; PP, pentose phosphate; z-score, expression z-score from CMap.

The integration of spatial transcriptomics and drug perturbation data through SOAR can enhance the efficiency and precision of drug discovery processes (Materials and Methods). Many of the compounds identified in our drug discovery effectively suppressed malignant cell–specific gene expression patterns by targeting the PI3K/Akt/mTOR growth and proliferation pathway (Fig. 1E). The PI3K/Akt/mTOR pathway promotes tumor proliferation, invasion, and endocrine resistance, and it is one of the most frequently dysregulated pathways in cancer (*51*–*55*). To ensure a multifaceted evaluation of potential therapeutic agents, we adopted several stringent criteria. First, the identified compounds targeting the PI3K/Akt/mTOR pathway are highly ranked in terms of their ability to suppress gene sets enriched in malignant cells, as evident by their high enrichment scores (ES) in the heatmap from SOAR’s drug discovery module (Figs. 1E and 4A). A second criterion is that these compounds should have neutral or promoting effects on gene sets enriched in immune cell. This criterion minimizes the risk of interfering with the normal functions of immune cells, including immune-mediated tumor killing (Fig. 4A). Using cell type–specific differentially expressed genes (DEGs) for drug discovery can thus facilitate the identification of compounds that selectively target pathways highly expressed in cancer cells (Fig. 4D). A third criterion is further refining the genes used in drug discovery to those that exhibit spatial variability, as these genes likely exhibit expression differences between tumor and nontumor regions. This criterion ensures focusing on the genes affected by local microenvironment signals rather than those with uniform expression across the tissue (Fig. 4D). The drug perturbation network from SOAR shows the gene targets of the compounds of interest along with protein-protein interaction (PPI) network of the target cell type (Fig. 4A). This resulted from a fourth criterion to select compounds inhibiting the PI3K/Akt/mTOR pathway that can either perturb spatially variable DEGs of malignant cells or genes that interact with them, suggesting the ability of these compounds in disrupting tumor processes.

By applying these rigorous criteria, we identified compounds that not only inhibit a key cancer proliferation pathway but also support immune function and precisely target the spatially nuanced gene expression landscape of tumor cells. These findings underscore the potential of such compounds as promising therapeutic candidates for reducing cancer progression, illustrating the power of SOAR’s integrated analysis to uncover drugs that meet multiple, critical benchmarks for efficacy and selectivity.

#### ERBB2/3 and PI3K/Akt/mTOR as targets for breast cancer therapy

**Fig. 5.**
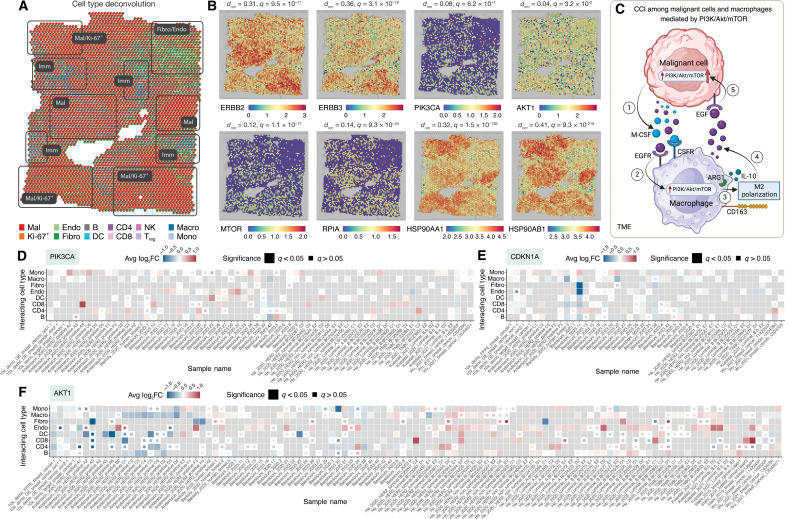
Uncover drug mechanism of action in cancer spatial transcriptomics data in SOAR. (**A**) Cell type deconvolution result of the breast cancer case study sample shows that there are distinct regions with high proportions of malignant and Ki-67^+^ cells, fibroblast and endothelial cells, and immune cells. (**B**) Spatial expression plots show regions with high *ERBB2/3*, *PIK3CA*, *AKT1*, *MTOR* expression overlap with regions that contain high proportion of malignant and Ki-67^+^ cells. *RPIA*, a downstream gene of *MTOR*, strongly down-regulated by the drug sirolimus, shows a similar expression pattern with *ERBB2/3*, *PIK3CA*, *AKT1*, and *MTOR*. Two-sided *t* tests were conducted to test whether the studied genes have different expression levels in capture locations with high (>50%) and low (≤50%) malignant/Ki-67^+^ proportions. The differences between normalized expression means and multiple testing–adjusted *P* values are shown. (**C**) Positive interaction between malignant cells and macrophages that is mediated by the PI3K/Akt/mTOR pathway. 1) Cancer cells release EGF and M-CSF into the TME, 2) where they bind to receptors on nearby monocytes and macrophages, activating the PI3K/Akt/mTOR pathway. 3) This activation increases expression of M2 macrophage markers (*CD163* and *ARG1*) and IL-10 secretion, fostering an immunosuppressive environment. 4) In response, M2 macrophages secrete more EGF and induce hypoxia, 5) further driving tumor progression. Neighborhood-based CCI results on breast cancer samples from SOAR shows that (**D**) *PIK3CA*, (**E**) *CDKN1A*, and (**F**) *AKT1* are often higher expressed in monocytes and macrophages when nearby cells are malignant cells, suggesting a positive interaction between the cell types. Abbreviations: CCI, cell-cell interaction; *d_nm_*, difference between normalized expression means; Imm, immune cells; TME, tumor microenvironment.

The PI3K/Akt/mTOR pathway is activated by the heterodimerization of HER2/3 following the binding of growth factors, which are overexpressed in the TME (Fig. 1E) (*52*, *54*, *55*). Following HER2/3 heterodimerization, a cascade of phosphorylation activates mTORC1, which up-regulates biosynthesis and metabolism and represses autophagy (*56*–*59*), as reflected from the DEGs of the breast cancer malignant cells (Fig. 1E). We observed higher expressions of PI3K, Akt, and mTOR as well as upstream regulators HER2/3 but not the tumor suppressor PTEN in regions with more malignant and proliferating Ki-67^+^ cells than nonmalignant cells (Figs. 1E and 5, A and B). Moreover, we observed significant spatial variability of *ERBB2* (encoding HER2) and *ERBB3* (encoding HER3) in malignant, endothelial, Ki-67^+^ cells and fibroblasts. Therefore, the expression of *ERBB2/3* and the downstream activation of PI3K/Akt/mTOR is context-dependent in tissue. Cancer cells secrete epidermal growth factors (EGF) and macrophage colony-stimulating factor (M-CSF) to the TME, which activate the PI3K/Akt/mTOR pathway in nearby monocytes and macrophages (Fig. 5, C to F) (*60*–*62*). The activated pathway, in turn, lead to elevated expression of M2 macrophage-associated markers *CD163* and *ARG1* as well as secretion of anti-inflammatory cytokine interleukin-10 (IL-10) (Fig. 5C) (*60*, *61*, *63*). M2 macrophages contribute to pro-tumorigenic outcomes via secretion of more EGF to the TME and other mechanisms like hypoxia induction (Fig. 5C) (*61*, *63*). Therefore, drugs inhibiting PI3K/Akt/mTOR have the potential to abrogate this positive feedback loop between proliferating malignant cells and monocytes as well as macrophages. In addition, PI3K/Akt inhibitors have also been shown to down-regulate *CXCL8* and *ATF3* (*64*). As a result, macrophages respond to the proinflammatory signals and switch toward an antitumoral M1 phenotype. In summary, SOAR’s spatial expression and variability as well as CCI analyses indicate that *ERBB2/3* and PI3K/Akt/mTOR are attractive drug targets for breast cancer cells due to their ability to mediate cell proliferation and modulate tumor immune microenvironment.

#### Potential anticancer agents targeting ERBB2/3 and PI3K/Akt/mTOR

SOAR’s drug discovery function can identify potential therapeutics that can precisely disrupt gene expression patterns and cellular interactions in regions saturated with proliferating malignant cells. Sirolimus is one such compound that can inhibit mTOR by interacting with FKBP12 (Fig. 1E) (*59*, *65*). SOAR’s drug perturbation analysis shows that sirolimus decreases the expression of biosynthesis genes, including those for glycolysis, pentose phosphate (PP), and lipid synthesis (Figs. 1E and 4B) (*57*, *58*, *66*–*68*). The most affected gene, RPIA, exhibited a spatial pattern where elevated expression overlapped with regions containing the highest concentration of Ki-67^+^ cells (Fig. 5B). This observation suggests that PP metabolism is likely a rate limiting factor for cell growth, and disrupting this process will reduce proliferation (*69*). In addition, sirolimus can also suppress mRNA translation, promote autophagy, and reduce mTORC2 activity to below the level needed to maintain Akt signaling (Fig. 1E) (*57*–*59*, *66*, *70*, *71*). The drug perturbation network from SOAR also showed that sirolimus’ analog everolimus down-regulates *ERBB2*, which participates in many PPIs related to cell growth, thereby reaffirming the drug’s ability to disrupt malignant cell proliferation (Fig. 4A). Sirolimus and everolimus have shown clinical efficacy in treating various solid tumors (*72*, *73*).

Trichostatin A (TSA), an antifungal antibiotic, is a class I histone deacetylase (HDAC) inhibitor and Akt inhibitor that also shows a strong suppression on malignant cells in SOAR’s drug discovery module (Figs. 1E and 4A). HDAC inhibitors have been evaluated in several clinical trials targeting solid tumors due to their ability to inhibit Akt phosphorylation and overcome PI3K/Akt treatment resistance through epigenetic modifications (Fig. 1E) (*74*–*78*). Specifically, TSA can acetylate the tumor suppressor p-53, resulting in the induction of apoptosis in malignant cells, as reflected from the up-regulation of apoptotic genes following TSA treatment in SOAR’s drug perturbation results (Figs. 1E and 4C). TSA can also inhibit cyclin-CDK–mediated cell cycle progression by down-regulating the overexpressed *CCND1* and *CDK4* and up-regulating their inhibitor *CDKN1A*, as reflected in SOAR’s perturbation network (Fig. 4, A and C) (*74*, *78*). In addition, *CDKN1A* expression in tumor cells, a signal for phagocytosis that can subsequently lead to antigen presentation (*79*), is elevated when nearby cells are monocytes, as shown from SOAR’s neighborhood-based CCI results (Fig. 5E). Therefore, by up-regulating *CDKN1A* in malignant cells, TSA could trigger monocytes differentiating into inflammatory macrophages. Through leveraging spatial data for drug discovery, SOAR was able to identify the repurposable drug TSA and showcase its ability to repress cell cycle progression in tumor cells.

Two compounds identified by SOAR’s drug discovery that can reduce HER2/3 activation are luminespib (AUY922) and BIBU-1361 (Fig. 1E). Luminespib is an inhibitor of the chaperon protein HSP90 that is required for Akt and HER2/3 stability (Fig. 1E) (*80*–*82*). HSP90 inhibitor leads to ubiquitination and degradation of HER2/3 and Akt, resensitizing HER2 tumors that were chemotherapy resistant due to the hyperactivation of PI3K/Akt/mTOR (*52*, *83*, *84*). As confirmed by spatial expression colocalization analysis, regions with high *HSP90AA1* and *HSP90AB1* also have higher levels of *ERBB2/3* (Fig. 5B). Therefore, perturbing the chaperone protein HSP90 will also perturb malignant regions that express high HER2/3, which HSP90 stabilize, limiting the response of these cells to growth factors in the TME. Luminespib has been studied for its broad-spectrum anticancer activity in more than ten clinical trials, including its application in ER-positive and HER2-positive breast cancer (*85*–*88*). Notably, in combination with trastuzumab for HER2-positive advanced or metastatic breast cancer, it demonstrated efficacy with a complete or partial response rate of 22% (*87*). The other compound that can inhibit HER2 activation, BIBU-1361, is a selective tyrosine kinase inhibitor that can block EGF-induced phosphorylation of EGFR (Fig. 1E). BIBU-1361 can suppress Akt activation, leading to reduced growth and increased apoptosis as shown from in vivo and in vitro studies (*89*, *90*). A shared target of luminespib and BIBU-1361 is the stimulatory alpha subunit of G protein (GNAS) (Fig. 4A). Following the binding of ligands to G protein–coupled receptors, GNAS binds with GTP, leading to increased production of cyclic AMP (*91*, *92*). As a result, various downstream signaling pathways associated with cell differentiation and metastasis are activated (*91*, *92*). Both luminespib and BIBU-1361 can down-regulate GNAS expression as shown by SOAR’s drug perturbation network and have the potential to mitigate the aberrant activation caused by the gain of function mutation of GNAS.

LY-294002 and wortmannin are two PI3K inhibitors identified by SOAR’s drug discovery function (Fig. 1E). LY-294002 treatment reversed PI3K/Akt-mediated abnormal activation of the multidrug resistance protein P-glycoprotein 1, anti-apoptosis proteins Bcl-2 and XIAP, and repression of apoptosis protein caspase-9 (*93*, *94*). LY-294002 treatment led to reduced Akt phosphorylation and GSK3 nuclear translocation, which corresponded to decreased transcription of the EMT marker *SNAI1* (encoding Snail) in a hepatocellular carcinoma model (Fig. 1E) (*95*). In addition, SOAR’s drug perturbation network shows that LY-294002 could restore the expression of down-regulated major histocompatibility complex class I antigens human leukocyte antigen (HLA)–DRA and HLA-DRB in malignant cells, thereby helping increase tumor recognition and infiltration by immune cells (Fig. 4A) (*96*). Wortmannin, a fungal metabolite, is another specific PI3K inhibitor with a similar mechanism of action, prioritized by SOAR’s drug discovery module (Fig. 1E) (*97*). Since both LY-294002 and wortmannin are competitive ATP binding PI3K inhibitors, both drugs are promising in disrupting the positive feedback loop involving proliferating cancer cells and monocytes (Fig. 5C). The increase of inflammatory M1 macrophages can potentially improve phagocytosis of tumor cells and antigen presentation to activated T effector cells (*98*, *99*). In summary, SOAR enables users to explore compounds that can reverse gene expression of pathological pathways and the mechanisms through which these compounds affect changes in cancer and other pathological samples.

#### Repurposing drugs to target inflammation in UC

**Fig. 6.**
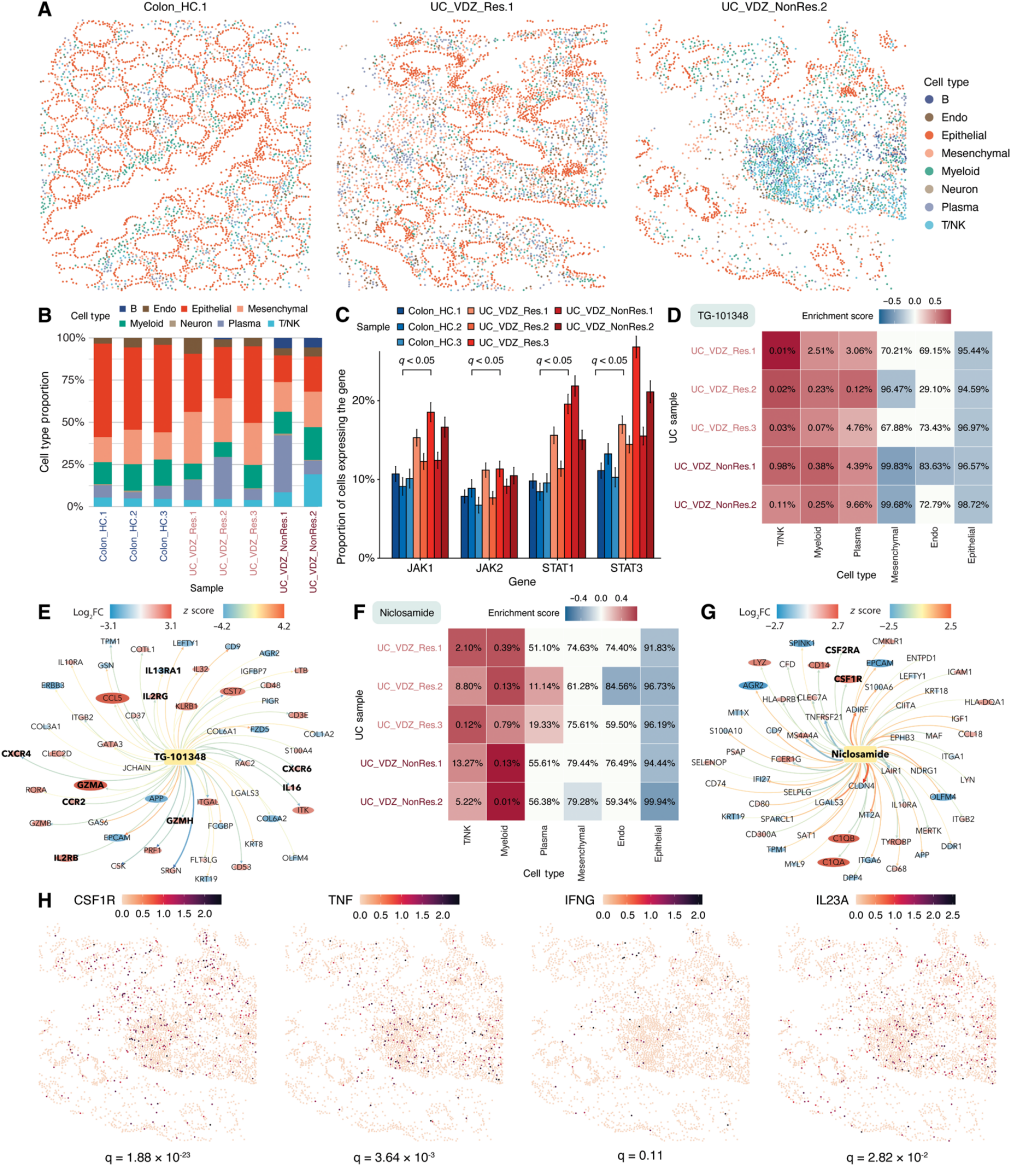
Spatial analysis and drug discovery show the repurposing potential of *JAK*/*STAT* inhibitors in UC. (**A**) Spatial distribution of annotated cells in healthy (Colon_HC.1), UC vedolizumab-responder (UC_VDZ_Res.1), and nonresponder (UC_VDZ_NonRes.2) colon tissue, highlighting epithelial destruction and immune cell infiltration in UC. (**B**) Nonresponders show increased T, NK, and myeloid cells (macrophages and monocytes) compared to healthy controls and responders. (**C**) *JAK*/*STAT* expression is elevated in patients with UC versus healthy controls (bars show 95% confidence intervals). (**D**) TG-101348 (federatinib) strongly suppresses spatially variable and DEGs in T, NK, and myeloid cells in UC samples without negatively affecting those of nonimmune cells. Heatmap percentages rank ES of TG-101348 among all tested drug perturbations. (**E**) TG-101348 down-regulates proinflammatory cytokine/chemokine receptors (*IL2RB*, *IL2RG*, *IL16*, *CCR2*, *CXCR4*, and *CXCR8*) and cytotoxic genes (*GZMB* and *GZMH*) in T and NK cells (bolded). Gene nodes are colored and sized by log-fold change in T and NK cells compared to other cell types; drug-gene edges are weighted by expression *z* scores from CMap. (**F**) Niclosamide strongly suppresses spatially variable and differentially expressed genes in myeloid cells without suppressive effects on those of nonimmune cells. (**G**) Niclosamide targets *CSF1R* and *CSF2RA* (bolded), upstream *JAK*/*STAT* activators. Gene nodes are colored/sized by log-fold change in myeloid cells compared to other cell types; drug-gene edges are weighted by expression *z* scores from CMap. (**H**) High *CSF1R*, *TNF*, *IFNG*, and *IL23A* expression aligns with myeloid, T, and NK cell–enriched regions. The two-sided Wilcoxon rank sum test results compare gene expression in these immune cells versus the others. Abbreviations: NonRes, nonresponder; Res, responder; UC, ulcerative colitis; VDZ, vedolizumab.

UC is a chronic inflammatory bowel disease (IBD) marked by persistent inflammation and ulceration of the colonic mucosa (*100*–*102*). UC arises from dysregulated immune responses to gut microbiota in genetically susceptible individuals, leading to excessive trafficking and/or activation of immune cells in the colon (*100*). This drives the overproduction of proinflammatory cytokines, such as tumor necrosis factor–α (TNF-α), interferon-γ (IFN-γ), and IL-23, which sustain inflammation and tissue damage (*101*). We conducted a comprehensive spatial and drug discovery analysis on published colon biopsy samples from a cohort of healthy controls (*n* = 3) and patients with UC receiving vedolizumab (*n* = 5), sequenced using CosMx (*103*). Hallmarks of UC pathogenesis include epithelial destruction, immune cell infiltration, and fibroblast expansion, as evident from comparing cell type distribution of a healthy participant and patients with UC (vedolizumab-responder and vedolizumab non-responder) (Fig. 6, A and B) (*103*).

Traditional treatments for UC have focused on either targeting individual cytokines or preventing immune cells from trafficking to the colon (*101*). Although these therapies have been proven to be effective in some patients, overall response rates remain low at approximately 30 to 40%, leaving an unmet medical need (*104*). The JAK-STAT pathway transduces signals from multiple cytokines to regulate the transcription of genes involved in immune activation and inflammation (*101*, *105*). Components of the JAK-STAT pathway exhibit increased expression in UC samples compared to healthy controls (Fig. 6C). The aberrant activation of JAK-STAT in macrophages and T cells exacerbates the inflammatory cascade and makes the pathway an optimal target for UC therapy.

SOAR’s drug discovery function finds small molecules targeting the JAK-STAT pathway, which offer a broader approach by modulating multiple proinflammatory, cytokine-dependent pathways (*101*, *106*). Among these, tofacitinib is a Food and Drug administration (FDA)–approved JAK inhibitor that demonstrated efficacy for treating UC in clinical trials and practice (*100*, *102*). SOAR’s drug discovery module ranks tofacitinib in the top 1% of all drug perturbations targeting T and NK cells across all vedolizumab-treated patients including nonresponders based on its drug ES. In addition, tofacitinib is also ranked within the top 1% of all drug perturbations targeting myeloid cells (including macrophages and monocytes) in most patients (fig. S4; see the Supplementary Materials). Recent case series have demonstrated that the combination of tofacitinib with vedolizumab may improve overall clinical effectiveness, and a large clinical trial is ongoing to study this combination (*107*, *108*). This result demonstrates that SOAR can identify potential drugs for nonresponders to conventional biologic therapy and help guide the potential design of future combination therapy clinical trials.

SOAR’s drug discovery function identifies two additional JAK-STAT inhibitors as strong UC repurposing candidates due to their strong suppression effects on T and myeloid cells. These drugs exhibit even less cytotoxic effects on noninflammatory cell types (e.g., endothelial cells) than tofacitinib (fig. S4 and Fig. 6, D to G).

SOAR identifies TG-101348 (federatinib), a selective JAK2 inhibitor approved for myelofibrosis, as strongly suppressing spatially variable, DEGs in T cells across all five UC samples (Fig. 6D). This result includes two samples that showed nonresponse to vedolizumab and thus would most benefit from alternative treatments or combination treatments. *IL2RG* and *IL2RB*, receptor subunits of the IL-2R complex, are up-regulated in T cells of UC samples and activate the JAK-STAT signaling pathway upon binding with IL-2 (Fig. 6E) (*109*). SOAR shows that TG-101348 can down-regulate these genes; therefore, this drug exhibits modulating effects on IL-2 overexpression and the downstream recruitment of immune cells. SOAR also reveals that TG-101348 down-regulates other cytokine and chemokine receptor genes linked to UC, including *IL16*, *CCR2*, *CXCR6*, and *CXCR4*, all of which play roles in T cell recruitment and inflammatory responses (Fig. 6E) (*101*, *105*). In contrast, SOAR identifies that *IL13RA1*, a gene encoding an IL-13 receptor subunit that suppresses inflammation, is down-regulated in UC samples but can be up-regulated by the drug (Fig. 6E) (*110*). Lastly, SOAR shows that *GZMA* and *GZMH*, cytotoxic T cell markers overexpressed in patients with UC, can be suppressed by the compound, potentially reducing epithelial barrier damage (Fig. 6E) (*101*, *105*). SOAR’s drug perturbation results with TG-101348 collectively demonstrate that JAK inhibitors can modulate multiple dysregulated inflammatory mechanisms in T cells of UC colon.

SOAR also identifies niclosamide, an FDA-approved anthelmintic drug and potent small-molecule STAT3 inhibitor, as a promising repurposing candidate for treating UC (*111*). Higher expression of *STAT3* and *STAT1* is observed in patients with UC, where the encoded proteins form heterodimers in response to IFN-γ binding to JAK1 and JAK2 (Fig. 6C) (*101*).The increased *STAT1* expression is largely driven by the infiltration of neutrophils and monocytes/macrophages (*106*). SOAR’s drug discovery module shows that niclosamide exhibits strong suppression toward spatially variable, DEGs in myeloid and T cells, with the strongest effect observed on myeloid cells of vedolizumab nonresponders (Fig. 6, A, B and F). Preclinical studies have investigated colon-targeted niclosamide delivery for IBD and found that it reduced disease activity index (*112*). Clinical trials have also shown promising results, achieving remission in 59% of participants with UC (*113*, *114*). Mechanistically, niclosamide down-regulates the CSF receptors (encoded by *CSF2RA* and *CSF1R*), which, when bound by their ligands, trigger the JAK/STAT signaling cascade (Fig. 6G) (*105*, *106*). SOAR’s spatial variability analysis shows that these CSF receptors, along with other JAK/STAT activators encoded by *TNF*, *IL23A*, and *IFNG*, exhibit spatially variable expression patterns that align with regions rich in myeloid cells (Fig. 6H) (*101*, *105*, *109*). These results highlight the power of SOAR’s drug discovery function in identifying and evaluating targeted therapeutics that modulate key inflammatory pathways and immune cell activity across various diseases such as UC and breast cancer.

## DISCUSSION

To facilitate data exploration and mega-analysis in spatial transcriptomics research, we present SOAR, a comprehensive spatial transcriptomics platform hosting systematically processed and annotated datasets. SOAR is beyond a data repository, offering a wide range of interactive analysis functions. It empowers users to visualize spatial gene expression, evaluate gene spatial variability, study neighborhood-based and distance-based CCIs, and identify potential drug candidates for treating pathological conditions.

Our case studies demonstrate SOAR’s practical utility by revealing spatial gene expression patterns and identifying CCIs in breast cancer samples. For example, we highlight the distinct proinflammatory and pro-tumor roles of *CXCL16* and *SPP1* in the TME. This illustrates that users may identify biologically relevant insights through SOAR’s spatial variability, CCI analysis functions, and visualization tools. By jointly analyzing spatial transcriptomics and drug perturbation data, SOAR’s drug discovery function unveils compounds that could suppress the PI3K/Akt/mTOR growth and proliferation pathway in patients with breast cancer, offering insights into drug repurposing opportunities and personalized therapy development. We also identified molecules targeting the JAK/STAT signaling pathway that can modulate multiple cytokine-dependent pathways in patients with UC, providing therapeutical options to those unresponsive to corticosteroids or therapies targeting individual cytokines. SOAR is thus a powerful platform that facilitates systematic comparisons across samples, cell types, and disease types, fostering biological insights and drug discoveries using spatial transcriptomics data.

Existing spatial transcriptomics platforms are often limited in scale, analytical capabilities, and potential translational applications. Most resources focus on basic functionalities such as spatial visualization, clustering, and variability analysis, with only a few supporting CCIs studies or cross-dataset mega-analysis. In addition, none of the current platforms provide tools for drug discovery, restricting their potential for therapeutic advancements. Compared with existing spatial transcriptomics platforms, SOAR offers unique advantages in both scale and functionality. SOAR combines extensive data collection with a wide range of interactive analysis functions, enabling systematic mega-analysis across different samples, cell types, and disease conditions. Furthermore, the inclusion of drug discovery capabilities sets SOAR apart as it bridges the gap between spatial transcriptomics data and translational applications. These differences highlight SOAR’s role as a versatile tool for researchers seeking to explore spatial transcriptomics data and derive therapeutic insights.

As the field of spatial transcriptomics continues its rapid evolution, we envision potential future improvements of SOAR. For example, SOAR can expand its scope by encompassing data from more types of species, tissues, and technologies, enriching our understanding of spatial gene expression across different biological systems. In particular, the integration of multimodal spatial omics data, such as spatial metabolomics, will enable users to gain a holistic view of genetic landscapes underlying various traits and disorders and further improve the robustness of SOAR’s drug discovery tools.

To our knowledge, SOAR is a novel spatial transcriptomics platform providing drug discovery results for different disorders and currently hosts the largest number of spatial transcriptomics samples with spatial coordinates data. SOAR is committed to continuous maintenance, improvement, and accessibility, ensuring its status as an evolving, open-access platform that meets the needs of the biomedical and clinical research communities. SOAR is updated on a quarterly basis, following a standardized pipeline including identifying newly published spatial transcriptomics datasets, systematically processing the data to ensure consistency and usability, conducting downstream data analyses, and updating the platform with harmonized datasets and integrated analysis results. With its scalable utilities, SOAR is geared to assist researchers in fully leveraging the potential of spatial transcriptomics for scientific discoveries and advancements.

## MATERIALS AND METHODS

### Data collection

We queried the Gene Expression Omnibus (GEO; http://ncbi.nlm.nih.gov/geo/) for human and mouse spatial transcriptomics datasets using the keywords “spatial+transcriptomics,” “spatial+transcriptome,” “spatial+RNA-seq,” and “spatial+RNA + sequencing” and downloaded 1156 datasets from unique GEO series (GSE) accessions. In addition, we manually reviewed the papers in the Museum of Spatial Transcriptomics (*10*) and collected 75 publicly available datasets. We also collected 115 datasets from other resources including Single Cell Portal (https://singlecell.broadinstitute.org/single_cell), 10x Genomics spatial gene expression demonstration datasets (https://support.10xgenomics.com/spatial-gene-expression/datasets), Spatial Research Lab (https://spatialresearch.org/resources-published-datasets/), 10x Genomics spatial publication list (https://10xgenomics.com/resources/publications), ALS-ST (*13*), spatialLIBD (*11*), STAR-FINDer (*12*), and Brain Research through Advancing Innovative Neurotechnologies Initiative—Cell Census Network (https://biccn.org/data). Next, we removed the duplicative datasets, validated that the downloaded data used next-generation sequencing or imaging-based spatial transcriptomics technology, and excluded the datasets missing spatial coordinates information.

In total, we have collected 441 datasets containing 3461 spatial transcriptomics samples from 19 different technologies and 13 species (human, mouse, rat, crab-eating macaque, chicken, pig, drosophila, canine, axolotl, zebrafish, thale cress, aspen, and chimera between mouse and human). The human and mouse samples come from different organs (bladder, brain, breast, digits, heart, intestine, joints, kidney, lacrimal gland, liver, lung, lymph node, mouth, muscle, nasopharynx, oral mucosa, ovary, pancreas, prostate, skin, spinal cord, spleen, stomach, testis, thymus, thyroid, and uterus) and other tissues including abdomen, body fat, bone, carotid artery, head and neck, scalp, the central nervous system, tumor cell lines, xenograft, and embryonic tissues.

### Data processing

We downloaded the count matrices and coordinate information for each dataset and applied a systematic data processing workflow to all the collected datasets. To account for the resolution and sequencing depth difference among spatial transcriptomics techniques, samples measured using different technologies were processed with corresponding quality control (QC) protocols. For 10x Visium, ST (*115*), sci-Space (*6*), CBSST-Seq (*116*), spatial-CITE-seq (*117*), DBiT-seq (*118*), Decoder-seq (*119*), MAGIC-seq (*120*), and Open-ST (*121*) datasets, we removed the capture locations with fewer than 500 unique molecular identifiers (UMIs), fewer than 500 genes, or ≥25% mitochondrial reads (*122*). We further excluded the capture locations with a total UMI count (or a total number of genes) three SDs below the median (*123*). Last, we filtered out the genes that are expressed in less than five capture locations. Single-cell-resolution technologies like 10x Xenium, CosMx (*124*), MERFISH (*125*, *126*), EEL-FISH (*127*), Stereo-seq (*128*), STARmap (*129*), osmFISH (*130*), seqFISH (*125*, *131*, *132*), and seqFISH+ (*133*) typically measure a smaller number of genes at lower sequencing depth. Therefore, we only performed cell QC on these datasets by removing capture locations with fewer than 500 UMIs or ≥25% mitochondrial reads (*122*). We performed QC on Slide-seq (*134*, *135*) samples so that the genes with total UMI counts less than 300 were excluded (*15*). In addition, only the capture locations with total UMI counts greater than 100 and less than 25% mitochondrial reads were included (*15*). Figure 1C demonstrates the average number of post-QC genes per sample, and fig. S1 shows the average number of UMI counts and genes per capture location of samples after QC in data generated by different technologies. After QC, we normalized and transformed the raw datasets using SCTransform, a framework for the normalization and variance stabilization of molecular count data (*136*). We next performed principal components analysis on normalized data and clustered the capture locations through a shared nearest neighbor approach (*123*). All data processing was conducted using R v4.1.1 and Seurat V4 (*123*). The processed samples were stored in a standardized format including an expression table (gene by capture location) and a coordinates table (capture location by Cartesian coordinates).

### Spatial clustering

Spatial clustering has been shown to identify spatial domains than ordinary clustering methods more accurately through jointly analyzing coordinates information and gene expression data (*137*). We performed spatial clustering using STAGATE (*137*), a graph attention auto-encoder framework for identifying spatial domains by learning low-dimensional latent embeddings from integrated spatial information and gene expression. STAGATE uses an attention mechanism to adaptively learn the similarity of neighboring spots (*137*). The radius cutoff is optimized for each spatial transcriptomics technology to achieve at least five neighbors per spot. STAGATE also incorporates a cell-type–aware module by pre-clustering gene expression to enhance characterization at spatial domain boundaries (*137*). The normalized counts of the top 3000 highly variable genes along with spatial locations are used to generate STAGATE embeddings. Clustering on the spatial embeddings is achieved via the Louvain algorithm, with the resolution parameter determined using the cell count of each sample (*123*).

### Cell type deconvolution and annotation

To perform cell typing, we curated reference single-cell RNA sequencing (scRNA-seq) datasets of different tissue types. We queried the GEO and identified scRNA-seq datasets with annotated cell types for each tissue type featured in SOAR. Next, we processed these datasets using an approach similar to that of the spatial transcriptomics dataset, including QC, normalization, and transformation.

In spatial transcriptomics data generated by certain technologies like 10x Visium, each capture location may contain multiple cells (*138*). Therefore, to perform accurate cell typing, deconvoluting the cell types of each capture location is needed (*138*). We performed cell type deconvolution on SOAR’s multiple cell–resolution spatial transcriptomics datasets using BayesPrism, a Bayesian method for predicting cellular composition and cell type–specific gene expressions in bulk RNA-seq and spatial transcriptomics data (*139*, *140*). To reduce batch effects, we excluded chromosomes X and Y, ribosomal, and mitochondrial genes from the analysis (*139*). The genes with expression greater than 1% of the total reads in more than 10% of capture locations were considered outlier genes (*139*) and were also removed. To improve cell typing accuracy, we only used the cell type signature genes for deconvolution analysis (*139*). The cell type markers were identified on the basis of the differential expression analysis results on the scRNA-seq reference. The predicted cell type fractions with above 0.5 coefficient of variation were clipped to zero to reduce noise (*139*, *140*). We further transformed the cell type–specific expression matrices into pseudo cell–level by dividing the expression values by the predicted fractions of that cell type in different capture locations. The pseudo-cells with zero fraction of the considered cell type were discarded, each pseudo-cell was assigned the coordinates of its original capture location, and the data corresponding to different cell types was next combined. The created pseudo cell–level data were used in subsequent distance-based CCIs and differential gene analysis.

For the single cell–resolution spatial transcriptomics datasets, we performed cell type annotation using SingleR (*141*), a method capable of annotating the cells in spatial transcriptomics datasets (*142*, *143*) on the basis of their similarities to reference scRNA-seq datasets with known cell types. The scRNA-seq datasets were then used as references for annotating the cell types of spatial transcriptomic capture locations of the corresponding tissue type. In particular, for noncancer brain datasets, we adopted a heuristic-guided approach to improve the performance of cell type annotation. Two scRNA-seq datasets from the Allen Brain Map (https://portal.brain-map.org/atlases-and-data/rnaseq) were used as the references—the Human Multiple Cortical Areas SMART-seq dataset (for annotating human samples) and the Mouse Whole Cortex and Hippocampus dataset (for annotating mouse samples). Their cells were annotated as glutamatergic, GABAergic, or nonneuronal following the Common Cell Type Nomenclature (*144*). Firstly, we identified marker gene sets for each cell type and each species by performing differential gene expression analysis on the corresponding reference scRNA-seq dataset using Seurat V4 (*123*). Next, we used AUCell (*145*) to score the activity of glutamatergic, GABAergic, and nonneuronal gene sets at each capture location based on marker gene expressions. Capture location clusters in the sample can then be classified as neuronal or nonneuronal according to the sum of AUCell scores across capture locations (*145*). Last, we used SingleR (*141*) to annotate the neuronal clusters as glutamatergic or GABAergic on the basis of a filtered version of the reference dataset that only contained neuronal cells.

### Website development

SOAR is a comprehensive and user-friendly platform that aids the exploration and analysis of spatial transcriptomics datasets. SOAR was implemented using the R Shiny framework (R v4.2.1, Shiny v1.7.1) on an Apache2 HTTP server and is compatible with smartphones and tablets. The website consists of six functional components “Home,” “Data Browser,” “Gene & Cell Analysis,” “Drug Discovery,” “Download,” and “Help” (fig. S5A). The Home module includes an overview of SOAR, and users can search for a gene of interest in this module. In the Data Browser module, users can identify a sample of interest using different filters and visualize its spatial gene expressions (fig. S5B). To facilitate temporal searching, the data browser is also sortable by the publication year, and users may identify datasets published in a specific year using the filter function (fig. S5B). Upon searching for a gene on the homepage, users will land in the Gene & Cell Analysis module, which enables users to evaluate the spatial variability of genes in different tissues and assess possible CCIs (fig. S5C). Notably, users may view the analysis results across multiple datasets using this module, allowing systematic comparisons among different samples and cell types. The Drug Discovery module allows users to investigate and visualize each pathological sample’s differential gene expression, PPI, drug enrichment, and drug perturbation analysis results (fig. S5D). All the results and visualizations from user-performed analyses are downloadable. In the Download module, users can download all the curated gene expression data, coordinate data, metadata, and sample-wise analysis results. The Help page documents the website and includes a tutorial with step-by-step instructions for using the platform. SOAR is free and open to all users at https://soar.fsm.northwestern.edu/, and there is no login requirement.

### Data Browser module

To aid user-conducted analysis, we constructed a comprehensive data browser that is part of SOAR and contains the metadata for all included spatial transcriptomics datasets. For each dataset, detailed information includes the hyperlink to the corresponding publication; the spatial transcriptomics technology used; and sample information including the number of samples, the species, organ, tissue, and the disease state of the sample. Furthermore, we document the average number of capture locations and genes in each sample after QC. Our data browser allows users to quickly select samples of interest to visualize spatial gene expressions; view spatial clustering, shared nearest neighbor–based nonspatial clustering, and cell typing results; and perform spatial variability analysis via interactive figures and tables. All the generated figures and tables are easily downloadable to support customized and large-scale research projects.

### Gene & Cell Analysis module

The gene search bar on the homepage of SOAR allows users to query the results of these analyses for a specific gene of interest. Upon searching for a gene, SOAR directs the user to the Gene & Cell Analysis tab, which subsequently prompts the user to narrow down the list of datasets by tissue type and species. In this tab, SOAR allows users to perform three types of analyses—spatial variability, neighborhood-based CCI, and distance-based CCI. The visualizations will be dynamically generated upon user query. All the *P* values were adjusted for multiple testing using the false discovery rate (FDR) approach, and we assume statistical significance at an adjusted *P* value of q<0.05.

#### Spatial variability

Studying the spatial variation of gene expression is helpful for understanding cell migration and CCIs (*1*, *20*). To facilitate the characterization of the functional architecture of complex tissues, we identified genes with spatial patterns of significant expression variation using SpatialDE, a statistical method for detecting spatially variable genes (*20*). Spatial variability analyses were conducted across the whole tissue and in different cell types on all the samples. When evaluating cell type–specific spatial variability, the deconvoluted expressions in individual cell types were used for multiple-cell resolution data, and the expressions in cells of the considered cell type were used for single-cell resolution data. Last, we used SpatialDE to perform automatic expression histology analysis, which groups the significantly (adjusted *P* value <0.05 ) spatially variable genes into common spatial expression patterns (*20*). This may potentially reveal histological patterns based on gene coexpression (*20*).

#### Neighborhood-based CCI

Cells of different cell types may interact through cell-cell contact (*33*). Spatial transcriptomics enables us to study CCIs by investigating whether a gene’s expression in one cell type appears to be promoted or inhibited when in another cell type’s neighborhood. To identify possible CCIs, we investigated whether the gene expression levels in a query cell type ( $CT_{Q}$ ) are influenced by its neighboring interacting cell type ( $CT_{I}$).

For multiple-cell resolution datasets, the pseudo cell–level deconvoluted expression data were used, and pseudo cells originating from the same capture location were considered neighbors. For single-cell resolution datasets, the cells adjacent to each other were considered neighbors. Cell type $CT_{Q}$ pseudo cells were denoted as $CL_{Q}$ and cell type $CT_{I}$ pseudo cells as $CL_{I}$ . To evaluate neighboring interactions, we performed Wilcoxon rank-sum tests to test whether genes are differentially expressed in $CL_{Q}$ adjacent and nonadjacent to $CL_{I}$ using the FindAllMarkers function in Seurat V4 (*123*). If a gene G is more highly expressed in $CL_{Q}$ adjacent to $CL_{I}$ , $CL_{I}$ may promote the expression of G in $CL_{Q}$.

#### Distance-based CCI

Interactions between cells can occur beyond simple adjacency through the secretion of cytokines (*33*). We evaluated the levels of CCIs through different signaling pathways in the CellChatDB database (*146*) using COMMOT (*147*), an optimal transport–based approach. For multiple cell–resolution data, the pseudo cell–level deconvoluted expression data were used. To characterize the CCIs over different distances, we performed COMMOT analysis using short (500 μm), medium (1000 μm), and long (1500 μm) distance thresholds (*147*). We further identified and clustered the genes differentially expressed with respect to increased received signal through each signaling pathway using tradeSeq (*148*) and COMMOT (*147*). These genes may be potential downstream genes regulated by genes in the studied pathway (*147*).

Both neighborhood-based and distance-based analyses can be performed when assessing CCIs. Their results can act as complementary parts and provide insight into whether the interaction happens more frequently at cell contact or over a long distance.

### Drug discovery module

In the Drug Discovery module, users may explore the drug screening results in samples of interest using the pathological sample browser. Four analysis modules are available, including differential gene expression, PPI, drug enrichment, and drug perturbation network, providing dynamically generated visualizations for download. All the *P* values were adjusted for multiple testing using the FDR approach, and we assume statistical significance at an adjusted *P* value of q<0.05.

#### Differential gene expression

Using the FindAllMarkers function in Seurat V4 (*123*), we performed Wilcoxon rank-sum tests to test whether genes are differentially expressed in a certain cell type compared with other cell types. For multiple cell–resolution data, the pseudo cell–level deconvoluted expression data were used. The testing was limited to genes with at least 0.1 log fold difference between two cell type groups and detected in a minimum fraction of 0.1 cells in either group. Using DEGs in the downstream Drug Enrichment analysis ensures that compounds passing the selection will exhibit cell type–specific suppression.

#### Protein-protein interaction

For each cell type, we extracted the associated PPI modules using a maximum of 300 most significant DEGs. For cell types that have enough deconvolved spots to conduct spatial variability analysis, the genes were filtered to those that also show spatial variability for the cell type. The resulting genes were then mapped to our human protein-protein interactome, which included 351,444 unique PPIs among 17,706 proteins (*149*–*152*). Selecting compounds that can repress genes with many PPIs satisfies the criteria that the resulting compounds can disturb various cellular processes necessary to the cell type of interest.

#### Drug enrichment

To identify drug candidates with the potential for disrupting dysregulated processes in pathological samples, we conducted in silico drug repurposing by performing gene set enrichment analyses on the DEG sets associated with each cell type. We retrieved the Connectivity Map (CMap) L1000 dataset (*153*) (accessed in October 2023) and analyzed perturbation profiles of 27,669 compounds associated with gene expression changes in 12,328 genes. These chemical perturbagens were treated in various cell lines and doses for 6 hours, resulting in a total of 145,491 drug perturbation profiles. Enrichment analyses were performed on DEG sets that also show spatial variability for cell types with enough deconvolved spots for spatial variability analysis. The spatial variability criterion for genes helps to ensure that the compounds selected could target pathways that respond to signals heightened in the pathological regions of the tissue. The gene sets needed to contain at least ten significantly (adjusted *P* value <0.05 ) up-regulated (log fold change >0.5 ) or down-regulated (log fold change <−0.5 ) genes, and fewer than 2000 total significant genes. We computed an ES for each eligible DEG set and CMap L1000 signature using a method described previously (*154*–*156*)

$$\begin{array}{c} \mathrm{ES}=\left\{ \begin{array}{c} \;{\mathrm{ES}}_{\mathrm{up}}-ES_{\text{down}},\;\text{sgn}\left(ES_{\mathrm{up}}\right)\neq \text{sgn}\left(ES_{\text{down}}\right)\\ 0,\;\text{sgn}\left(ES_{\mathrm{up}}\right)=\text{sgn}\left(ES_{\text{down}}\right) \end{array}\right. \end{array}$$

The indices of the DEGs sorted by their ranks in the drug perturbation profiles in ascending order are denoted as j=1,2,…,s . Further, the total number of genes in the profile was denoted as r and the rank of genej as V(j) , where 1≤V(j)≤r . We calculate $ES_{\mathrm{up}}$ and $ES_{\text{down}}$ for the up-regulated DEGs and down-regulated DEGs separately

$$a=\;\underset{1\leq j\leq s}{\text{max}}\left(\frac{j}{s}-\frac{V\left(j\right)}{r}\right)$$

$$b=\;\underset{1\leq j\leq s}{\text{max}}\left(\frac{V\left(j\right)}{r}-\frac{j-1}{s}\right)$$

ESup={aup,aup>bup−bup,aup<bup

ESdown={adown,adown>bdown−bdown,adown<bdown

To calculate the one-tailed *P* values of the ES, permutation tests were repeated 10,000 times using randomly generated gene lists with the same n(up) and n(down) as the DEGs. Depending on the sign of the ES, the associated one-tailed *P* value measures either the left ( ES<0 ) or right ( ES>0 ) tail. When ES<0 , the drug is positively related to the selected differential expression comparison and may cause a change in gene expression similar to that in the cell type of interest. On the other hand, when ES>0 , the drug is inversely related to the comparison and may lead to opposite gene expression patterns. Selecting compounds with ES>0 in pathological cell types and ES<0 in nonpathological cell types ensures that these compounds can perturb pathological processes without affecting normal functions.

#### Drug perturbation network

For each set of drug enrichment analysis results, the top 500 inversely related ( ES>0 , adjusted *P* value <0.05 ) and top 500 positively related ( ES<0 , adjusted *P* value <0.05 ) drugs were used to generate the drug perturbation network (*154*–*156*). In the network, each node is a drug or gene, and each edge reflects an identified perturbation of the drug on the gene. The gene nodes are colored by their log fold changes in DEG analysis, and the edges are colored by the standardized drug perturbation scores.

#### Case study compounds selection criteria

For our breast cancer case study, we identified compounds that can inhibit the PI3K/Akt/mTOR pathway based on the following selection criteria. The compounds inversely related ( ES>0 ) to the genes that are both differentially expressed and spatially variable in malignant cells set were selected. Next, we further narrowed the compound list by selecting those positively or neutrally related ( ES≤0 ) to immune cell DEG sets. Using the drug perturbation and PPI networks with respect to malignant cells, we also ensured that the compounds could perturb the spatially variable DEGs or genes interacting with them. In total, seven compounds passed the criteria, including sirolimus, everolimus, TSA, luminespib, BIBU-1361, LY-294002, and wortmannin.

## Supplementary Materials

### The PDF file includes:

Website Manual Figs. S1 to S17 Legends for tables S1 and S2
