# Supplementary material for: SOAR elucidates biological insights and empowers drug discovery through spatial transcriptomics
Source: Sci Adv. 2025 Jun 11;11(24):eadt7450. doi: 10.1126/sciadv.adt7450 (PMC13109963; doi:10.1126/sciadv.adt7450)
Supplement: Supplementary file 1 — Website Manual Figs. S1 to S17 Legends for tables S1 and S2 [file sciadv.adt7450_sm.pdf]

Science Advances
AAAS

Supplementary Materials for

SOAR elucidates biological insights and empowers drug discovery through spatial transcriptomics

Yiming Li *et al.*

Corresponding author: Yuan Luo, yuan.luo@northwestern.edu; Zexian Zeng, zexianzeng@pku.edu.cn

Sci. Adv. **11**, eadt7450 (2025)
DOI: 10.1126/sciadv.adt7450

The PDF file includes:

Website Manual
Figs. S1 to S17
Legends for tables S1 and S2

Other Supplementary Material for this manuscript includes the following:

Tables S1 and S2

Website Manual

SOAR is a comprehensive spatial transcriptomics platform with biological insights exploration and drug discovery capabilities (**Fig. S6**). This manual will demonstrate the interactive analysis functions offered by SOAR.

Data Browser

When users click on the "Data Browser" tab, they can browse the 3,461 samples hosted by SOAR and filter for samples based on their research interests using the top menu (**Fig. S7**). Each row in the data browser table corresponds to one sample. Metadata is listed for each sample.

The following tutorial will be based on the first row, 10x_demo_GE_breast_cancer_sec1, a breast cancer sample. Upon clicking on the sample number, users can scroll down and view the spatial variability of expression in genes of interest (**Fig. S8A**). For example, we can enter the gene SPP1, which is a marker for tumor-associated macrophages that can help malignant cells avoid immune surveillance. This gene demonstrates high spatial variability at the bottom left and right corners of the tissue image, as evident from the color gradient patterns. The gene expression visualization is shown as spots overlayed on the original tissue image. The opacity of spots can be lowered if users are more interested in viewing the tissue morphology.

The table below the spatial gene expression visualization panel presents the spatial variability of gene expression across different cell types (**Fig. S8B**). Multiple gene inputs are allowed for easier comparison. For example, we can compare the spatial variability of SPP1 and CXC16, whose role is the opposite of SPP1, as it can recruit immune cells to sites of damage. Once sorted by q-value, we see that while CXCL16 is more uniformly expressed in dendritic cells and

monocytes, it shows significantly variable expression in other cell types depending on the cells' spatial location.

We can further leverage the "Cellular Deconvolution" tab where each spot is represented as a pie chart of the deconvoluted cell type percentages in that capture location to better understand the spatial variability results we observed (**Fig. S9**). For example, the bottom left and right corners that showed higher SPP1 expression indeed overlap with higher composition of malignant cells and macrophages from deconvolution. We can also see that the region above the bottom left corner are spots that contain more CD4 T cells.

To demonstrate the "Spatial Clustering" function, we will explore a different colon sample, Colon_HC.1 (**Fig. S10**). In this sample, we can see that there are 7 distinct spatial domains, representing different tissue structure, including epithelial lining around the crypt and the immune cells below epithelium. The "Clustering (non-spatial)" function provides an alternative way to explore clusters based solely on gene expression (**Fig. S11**). In the colon sample, we observed strong agreement between clustering results with and without spatial information. Both methods effectively identify clusters that correlate with functional properties.

Gene & Cell Analysis

The "Gene & Cell Analysis" module allows users to investigate the cell-type specific expression pattern and cell-cell interaction of specific genes. Upon entering a tissue and species, users can then select a gene of interest, such as SPP1.

The "Spatial variability" tab allows users to conduct mega-analysis of spatially variable genes across different samples (**Fig. S12**). Red tiles represent significant results whereas white represent non-significant results, and grey tiles mean gene expression is not measured in the cell

type. For example, we can see that SPP1 tends to have significantly spatially variable expression in endothelial and macrophages as well as fibroblasts and malignant cells but not in T or B or other myeloid cells. This result concurs with SPP1's role in promoting cell proliferation and migration.

The "Neighborhood-based cell-cell interaction" tab shows the differential gene expression analysis results in a cell type of interest located adjacent and non-adjacent to other cell types (**Fig. S13**). The significant results are shown as larger tiles. Red represents positive log fold change in expression, whereas blue represents negative log fold change. For example, we can see that SPP1 in macrophages has higher expression when they are adjacent to malignant cells.

The "Distance-based cell-cell interaction" tab models the relationship between the expression of a gene in a cell type and its distance to other cell types (**Fig. S14**). Significant results are shown as larger tiles. A red color indicates potential promotor effect of the interacting cell type. For example, SPP1 expression in macrophages shows the greatest positive relationship with their distance to malignant cells, fibroblasts, and endothelial cells. This result concurs with tumor-associated macrophages' role in promoting uncontrolled growth in these cells.

Drug Discovery

The "Drug Discovery" module allows users to identify compounds with potentially therapeutic effects for pathological samples contained within SOAR. The perturbation effects of these compounds on cells and genes have been experimentally tested by CMap from the Broad Institute.

The "Pathological Sample Browser" allows for filtering by tissues, conditions, and compounds of interest (**Fig. S15**). The enrichment score represents the overall perturbation effect a CMap

instance has on the differentially expressed genes of a cell type, which are filtered to be malignant cells in this example. Positive scores mean repression of differentially expressed genes and vice versa. The CMap instances can be mapped back to the published CMAP dataset for in-depth analysis on experimental conditions. Non-filtered results can be downloaded at the bottom.

The first row is the breast cancer sample we have been focusing on for the tutorial, and the compound everolimus is a repressor of the mTOR complexes. Sample number can be clicked to further investigate the specific sample and perturbation.

Before delving into specific compounds, we can first investigate the differentially expressed genes in malignant cells, for example, which can help to identify gene targets (**Fig. S16A**). We see that the MHC HLA genes are downregulated, aiding malignant cells to escape from immune surveillance. On the other hand, ERBB3 (or HER3), an upstream gene of mTOR, is upregulated.

In the right panel, we can examine the protein-protein interaction in the cell type of interest, which in this case is malignant cells (**Fig. S16B**). The node colors represent the degree of differential gene expression. We can see that ERBB3 interact with HLA genes. We can also observe that CCND1 (cyclin D1) interacts with its suppressor CDKN1A (cyclin-dependent kinase inhibitor 1A). High expression of CCND1 and low expression of its suppressor are markers of uncontrolled cell growth and differentiation.

In the bottom left panel, we can investigate the top and bottom 200 compounds in terms of enrichment scores on the cell type of interest, which in this case is malignant cells (**Fig. S17A**). Alternatively, if we are interested in compounds that can promote the expression of differentially expressed genes in immune cells, we may look at the positively enriched drugs list instead.

The bottom right panel allows us to investigate the gene targets of individual CMap instances, such as everolimus (**Fig. S17B**). Node colors represent the direction of log2 fold change of the gene, and arrow colors represent the direction of perturbation the compound has on the gene target. The top 30 and bottom 30 targets are shown. We can see that everolimus strongly promotes the expression of the downregulated CDKN1A gene in malignant cells, which can promote cell cycle arrest. Everolimus can also downregulate the upregulated MYC and SLC5A6, which are responsible for oncogenic metabolism. This diagram helps explain the strong suppression everolimus has on malignant cells by elucidating its mechanism of action.

Supplementary Figures

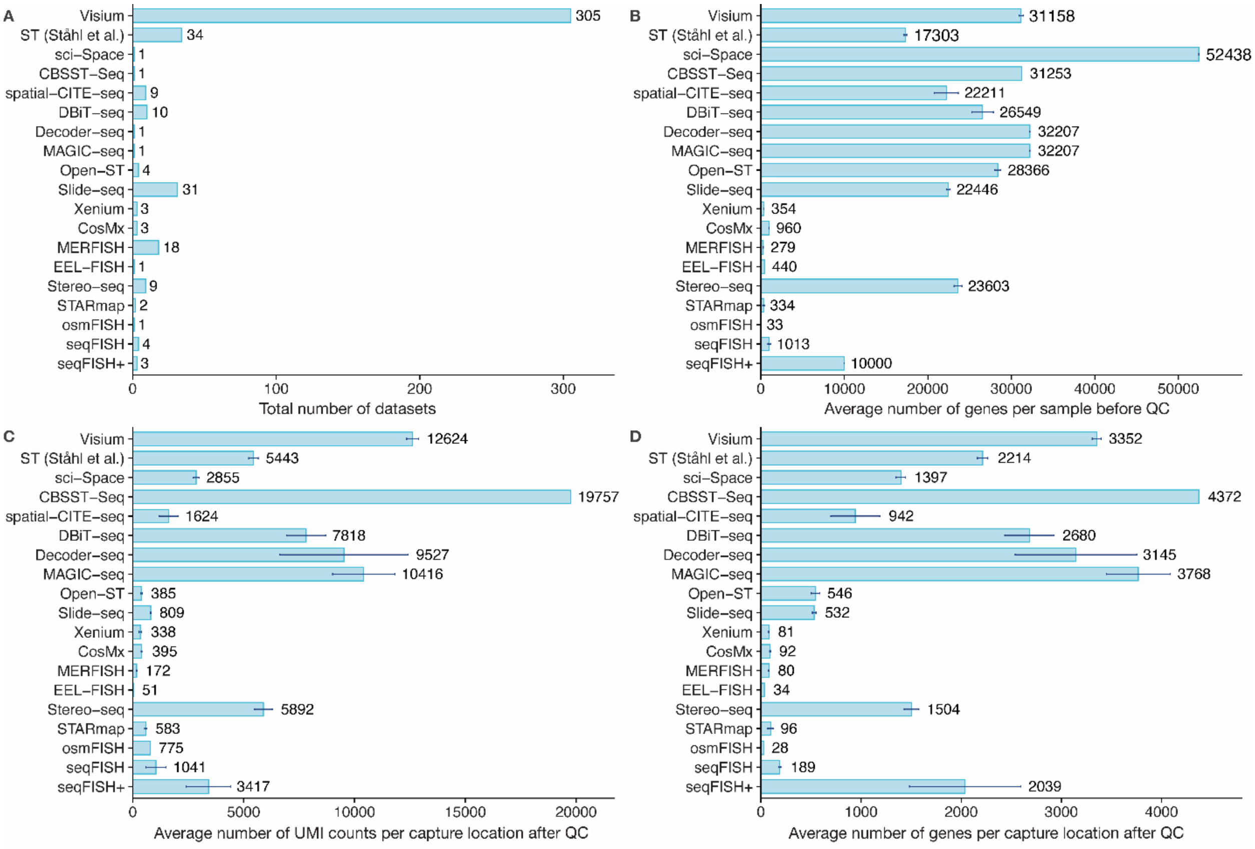

Fig. S1. Summary statistics of data from different spatial transcriptomics technologies. **(A)** The number of datasets, **(B)** the average number of genes per sample before quality control, as well as the average number of **(C)** UMI counts and **(D)** genes per capture location after quality control are shown. The 95% confidence intervals for the means are plotted as error bars.

Abbreviations: QC, quality control; UMI, unique molecular identifier.

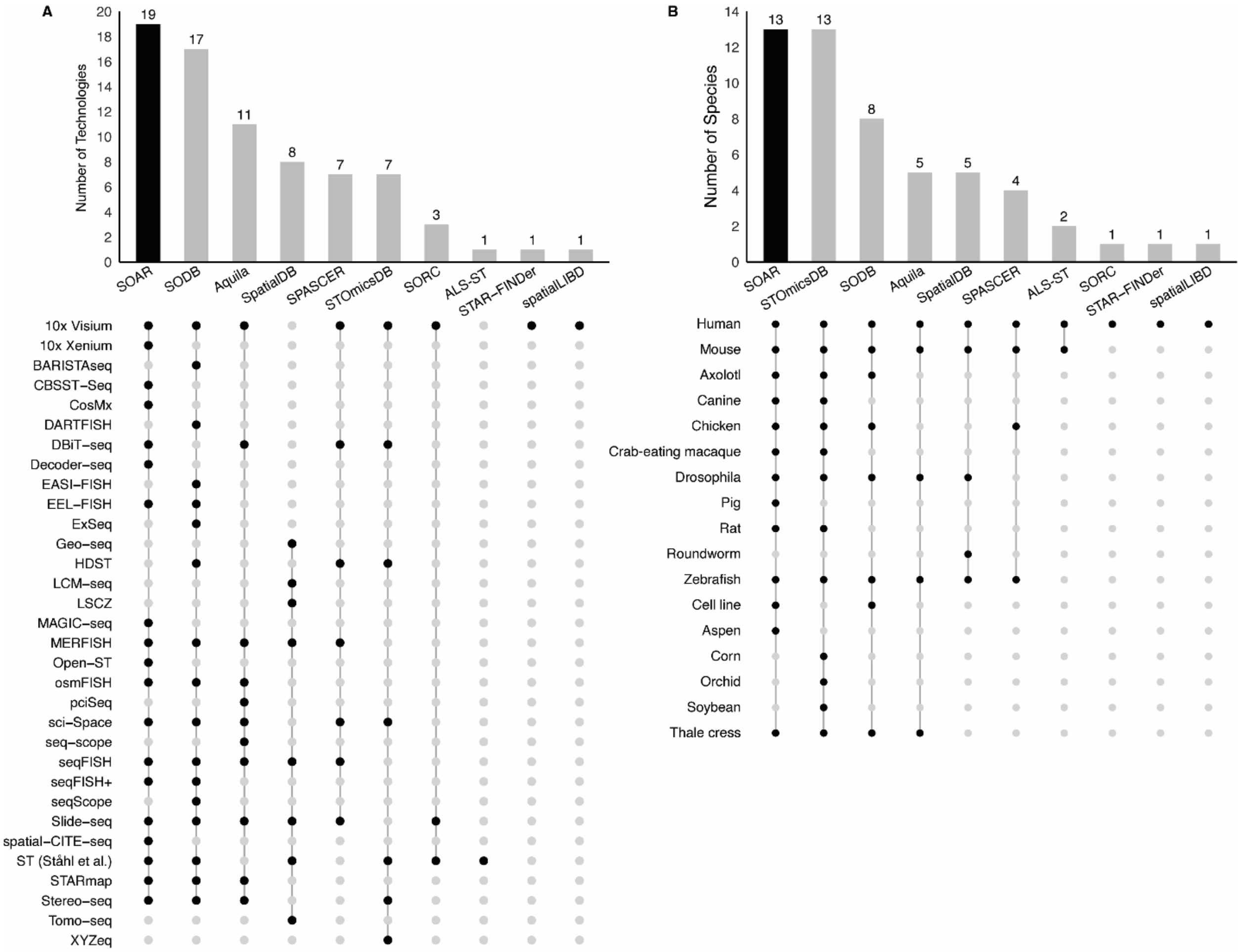

Fig. S2. The coverage of different spatial transcriptomics technologies and species in different spatial transcriptomics resources. **(A)** The number of technologies and **(B)** the number of species covered by different spatial transcriptomics resources are shown.

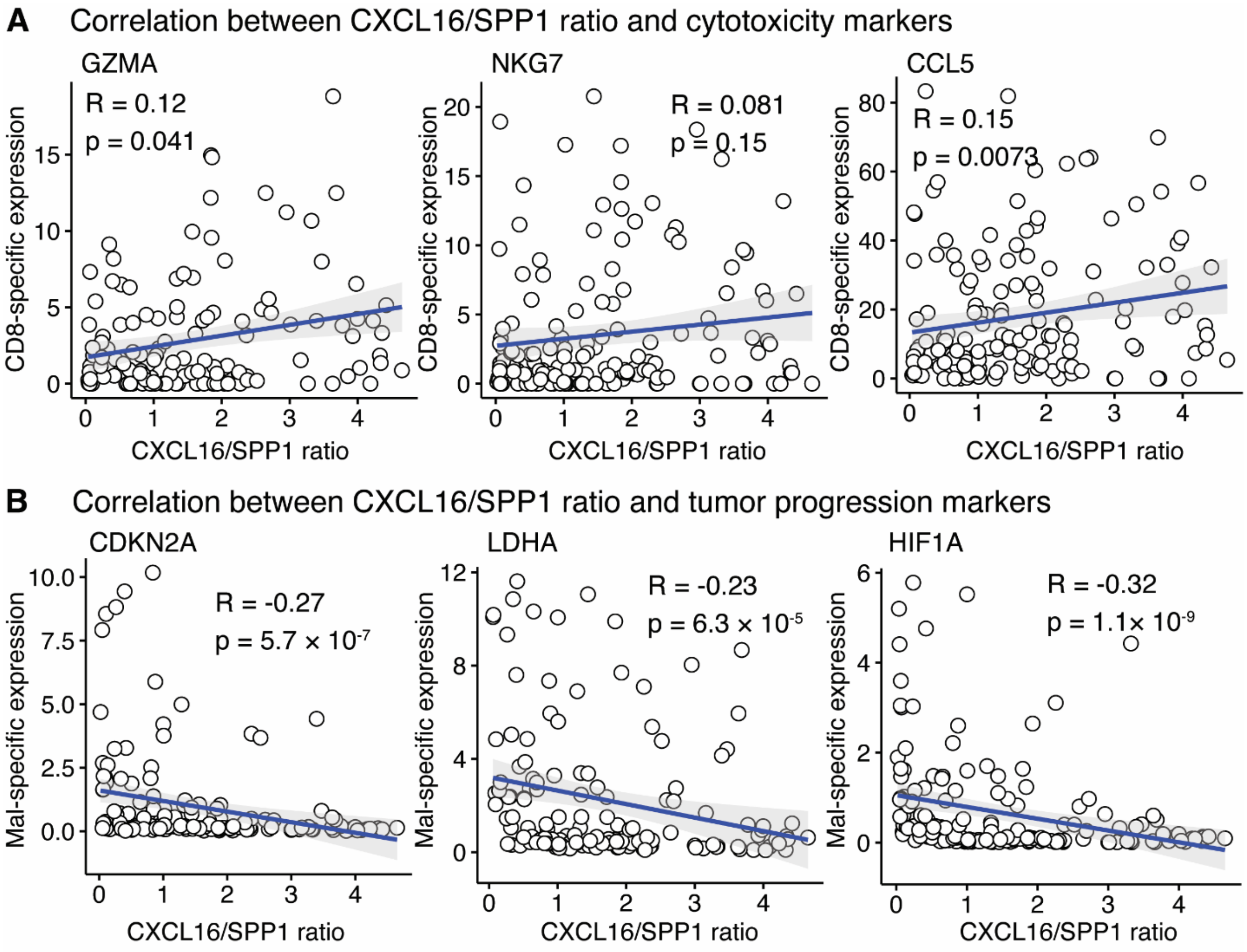

Fig. S3. CXCL16/SPP1 ratio in macrophages correlates with the expression of (**A**) cytotoxicity and (**B**) tumor progression markers in breast cancer samples. p-values are adjusted using the false discovery rate approach. **Abbreviations**: Mal, malignant cells.

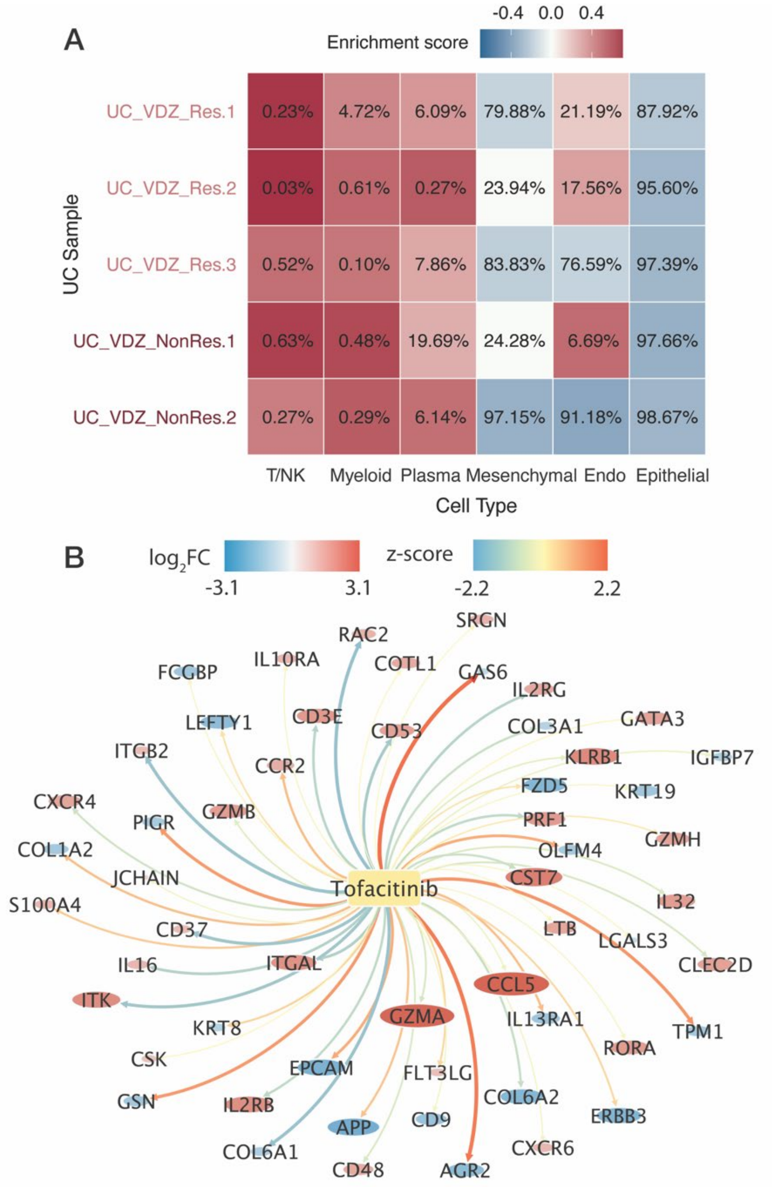

Fig. S4. **Drug enrichment and gene perturbation results of tofacitinib. (A)** Tofacitinib, a JAK inhibitor clinically approved for treating ulcerative colitis, showed strong suppression toward spatially variable and differentially expressed genes in T, NK, and myeloid cells in UC samples. This compound appears to have a mild repressive effect on endothelial cells of some samples. Heatmap percentages represent the drug's perturbation enrichment score ranked as a percentile among all tested drug perturbations. (**B**) Drug perturbation network of tofacitinib shows that it is capable of downregulating the chemokine receptors, IL2RB and IL2RG, and

cytotoxic gene, GZMA, that are higher expressed in T and NK cells of UC patient. The gene nodes are colored and sized by their log fold change in T and NK cells compared with other cell types. The edges between drugs and genes are colored and weighted by the expression z-score from Connectivity Map. **Abbreviations**: Endo, endothelial cells; log2FC, log-fold change; NK, natural killer cells; NonRes, non-responder; Res, responder; UC, ulcerative colitis; VDZ, vedolizumab; z-score, expression z-score from Connectivity Map.

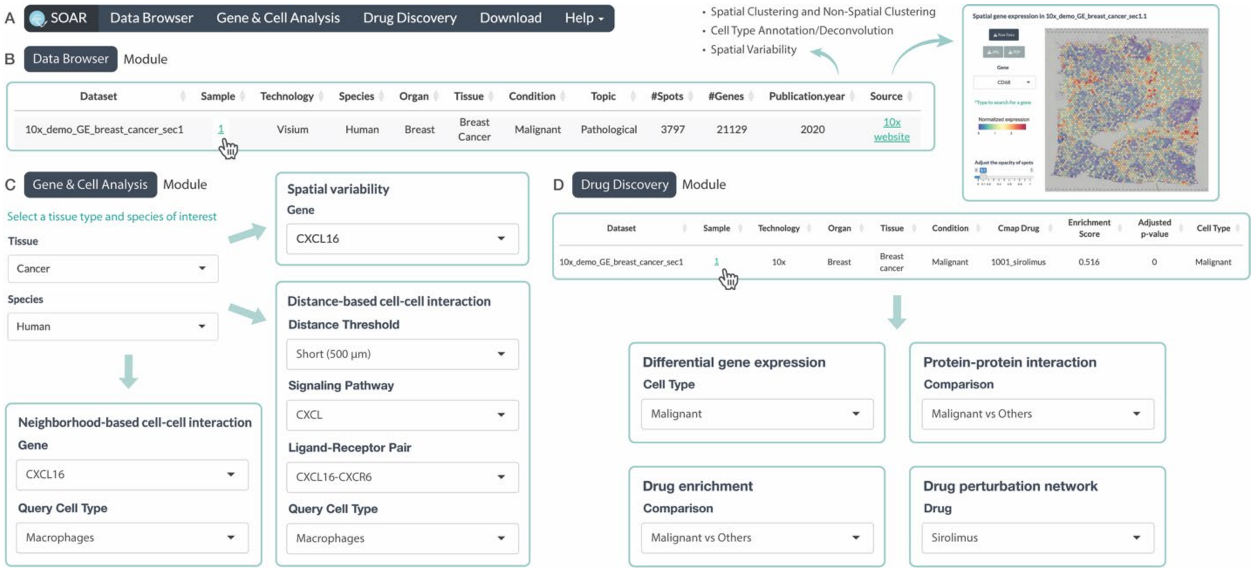

Fig. S5. Interactive interfaces of SOAR. (A) Main modules of SOAR, including “Data Browser”, “Gene & Cell Analysis”, “Drug Discovery”, “Download”, and “Help”. **(B)** In SOAR’s “Data Browser” module, users can identify a sample of interest and visualize its spatial gene expression. **(C)** In SOAR’s “Gene & Cell Analysis” module, users can first select a tissue type and species of interest. Next, users can perform spatial variability analysis or explore neighborhood-based and distance-based cell-cell interactions by interactively inputting a gene and/or query cell type. **(D)** In SOAR’s “Drug Discovery” module, users can identify a pathological sample of interest and perform differential gene expression, protein-protein interaction, drug enrichment, and drug perturbation network analysis.

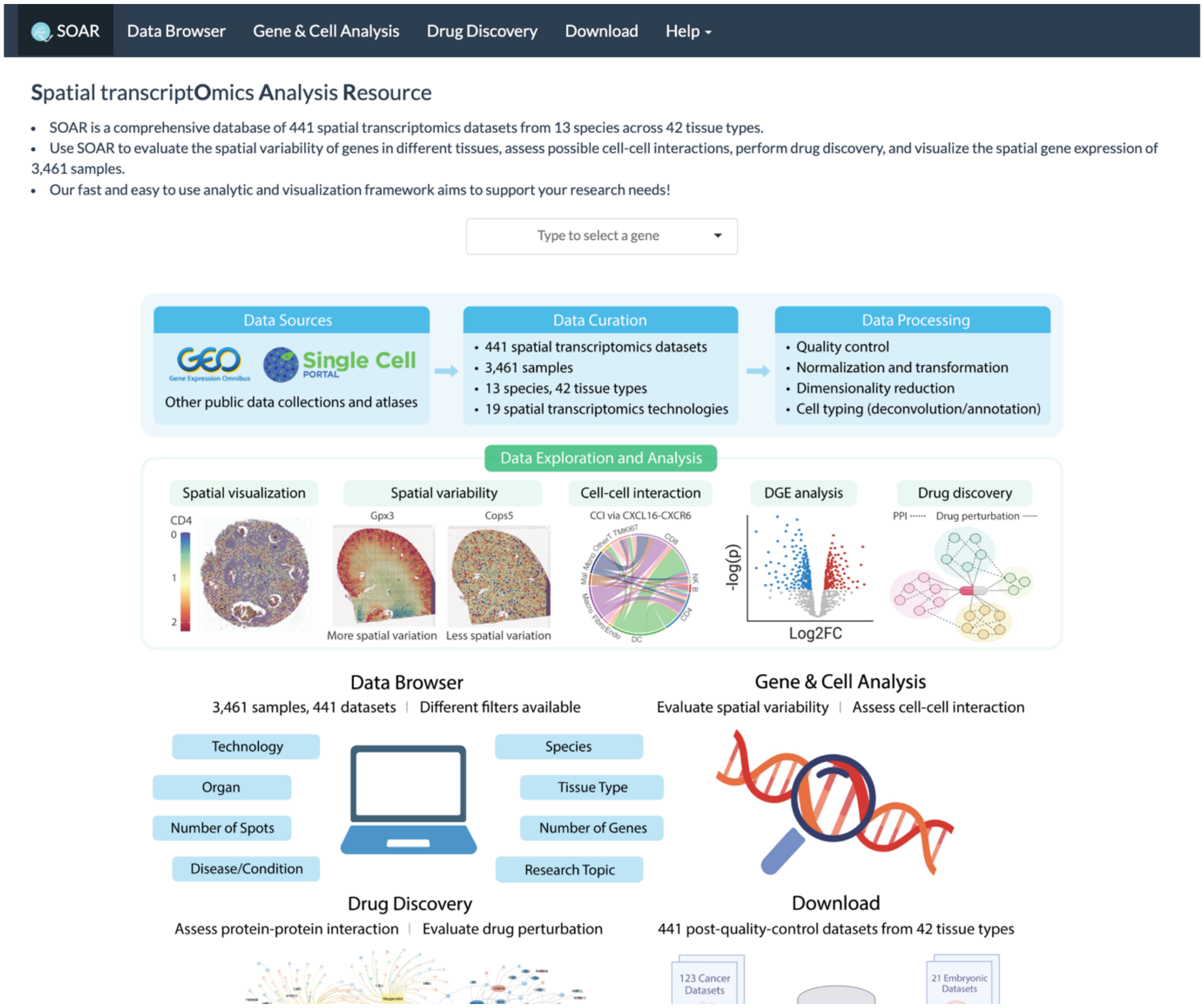

Fig. S6. Landing page of SOAR. The landing page provides an overview of the spatial transcriptomics datasets as well as analysis function as offered SOAR. Users can also enter a gene of interest to directly access the Gene & Cell Analysis module.

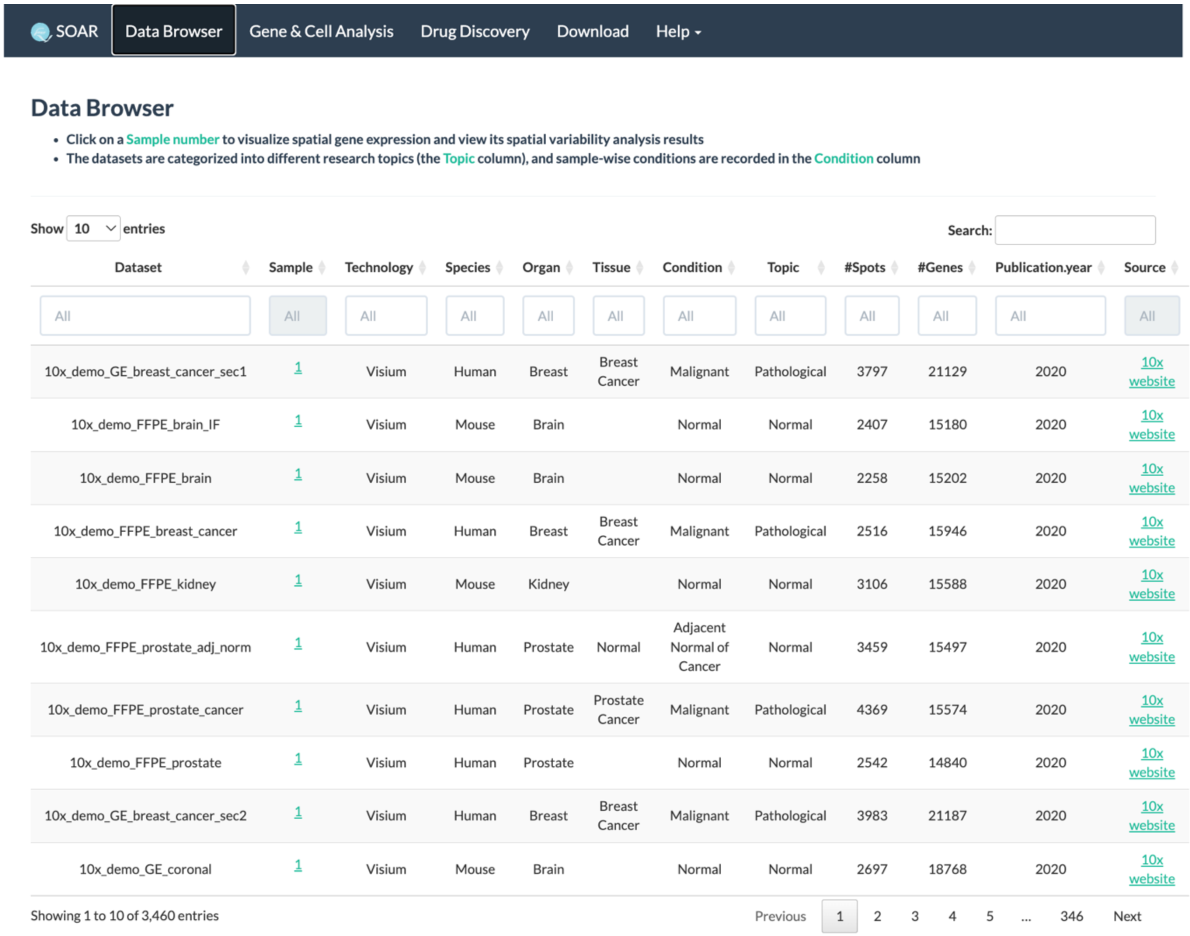
SOAR | Data Browser | Gene & Cell Analysis | Drug Discovery | Download | Help

Data Browser

- Click on a Sample number to visualize spatial gene expression and view its spatial variability analysis results
- The datasets are categorized into different research topics (the Topic column), and sample-wise conditions are recorded in the Condition column

Show 10 entries

Search:

Dataset	Sample	Technology	Species	Organ	Tissue	Condition	Topic	#Spots	#Genes	Publication.year	Source
All	All	All	All	All	All	All	All	All	All	All	All
10x_demo_GE_breast_cancer_sec1	1	Visium	Human	Breast	Breast Cancer	Malignant	Pathological	3797	21129	2020	10x website
10x_demo_FFPE_brain_IF	1	Visium	Mouse	Brain		Normal	Normal	2407	15180	2020	10x website
10x_demo_FFPE_brain	1	Visium	Mouse	Brain		Normal	Normal	2258	15202	2020	10x website
10x_demo_FFPE_breast_cancer	1	Visium	Human	Breast	Breast Cancer	Malignant	Pathological	2516	15946	2020	10x website
10x_demo_FFPE_kidney	1	Visium	Mouse	Kidney		Normal	Normal	3106	15588	2020	10x website
10x_demo_FFPE_prostate_adj_norm	1	Visium	Human	Prostate	Normal	Adjacent Normal of Cancer	Normal	3459	15497	2020	10x website
10x_demo_FFPE_prostate_cancer	1	Visium	Human	Prostate	Prostate Cancer	Malignant	Pathological	4369	15574	2020	10x website
10x_demo_FFPE_prostate	1	Visium	Human	Prostate		Normal	Normal	2542	14840	2020	10x website
10x_demo_GE_breast_cancer_sec2	1	Visium	Human	Breast	Breast Cancer	Malignant	Pathological	3983	21187	2020	10x website
10x_demo_GE_coronal	1	Visium	Mouse	Brain		Normal	Normal	2697	18768	2020	10x website

Showing 1 to 10 of 3,460 entries

Previous 1 2 3 4 5 … 346 Next

Fig. S7. SOAR's Data Browser module. Data Browser allows users to observe and filter by metadata as well as access the source of each sample. Users can click on Sample to access sample-wise analysis results.

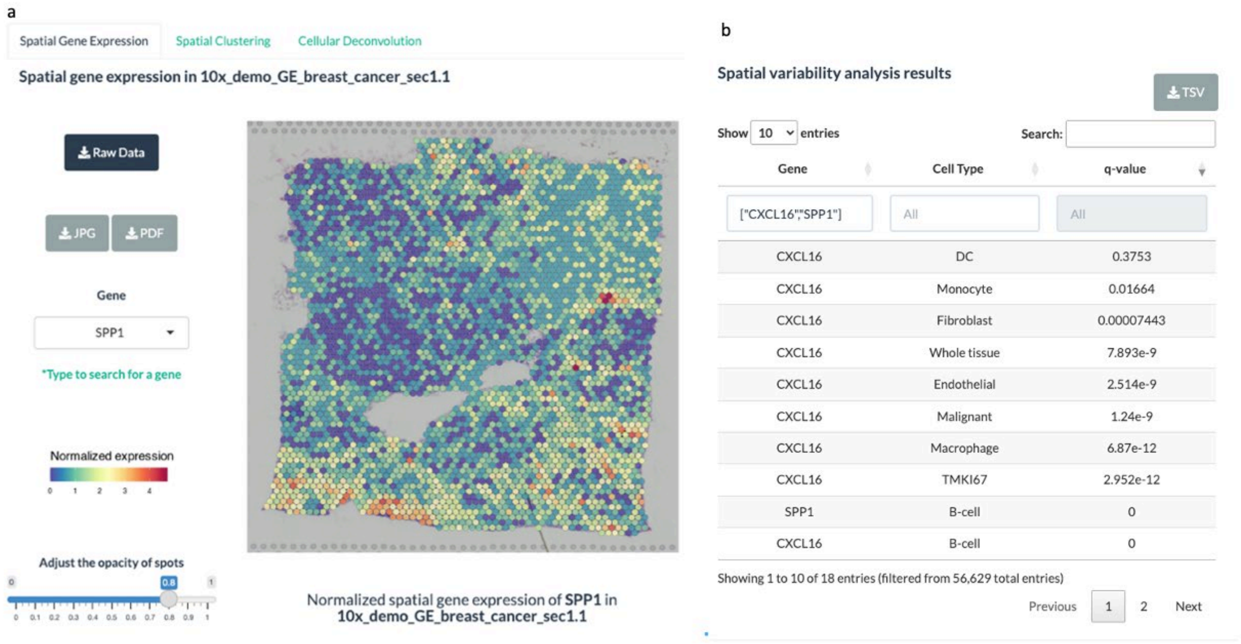

Fig. S8. Spatial gene expression visualization and spatial variability analysis functions in the Data Browser. (A) Upon entering a gene of interest, a user can view the expression of this gene across the spatial regions of a sample. **(B)** In the spatial variability analysis results table, users can view the significance of interested genes' spatial variability at the cell type and tissue level.

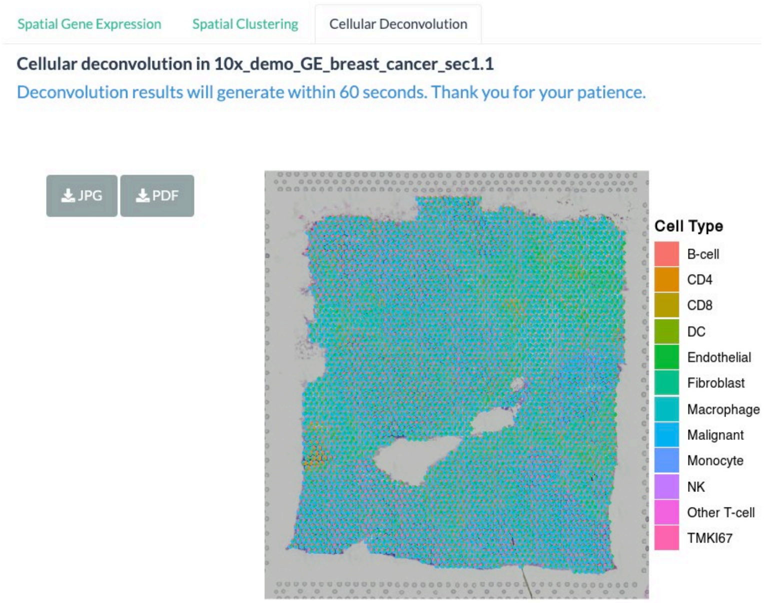

Fig. S9. Perform cellular deconvolution in SOAR's Data Browser. Deconvolution results are shown as pie charts of deconvolved cell types to reflect composition of each spot.

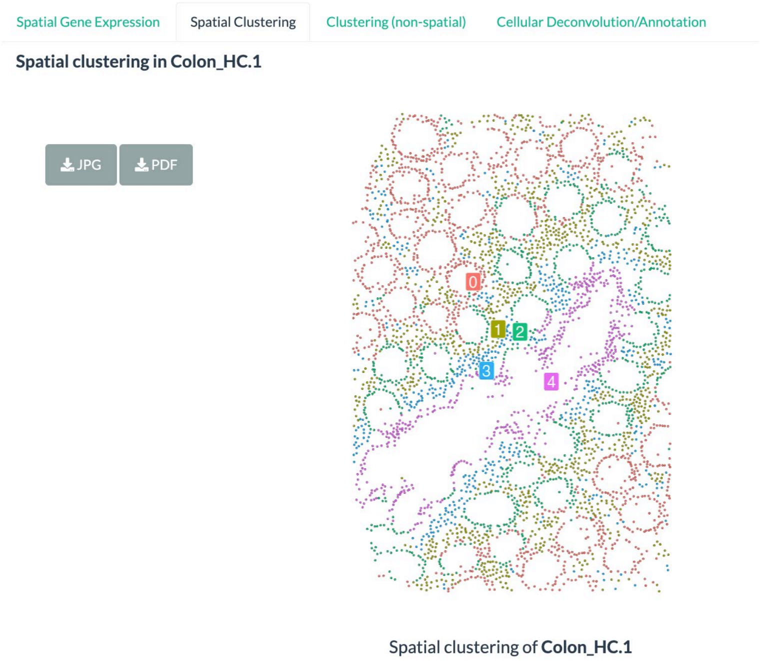

Fig. S10. Perform spatial clustering in SOAR's Data Browser. Spatial clustering on the colon sample shows clusters that correlate with tissue structure and functionality.

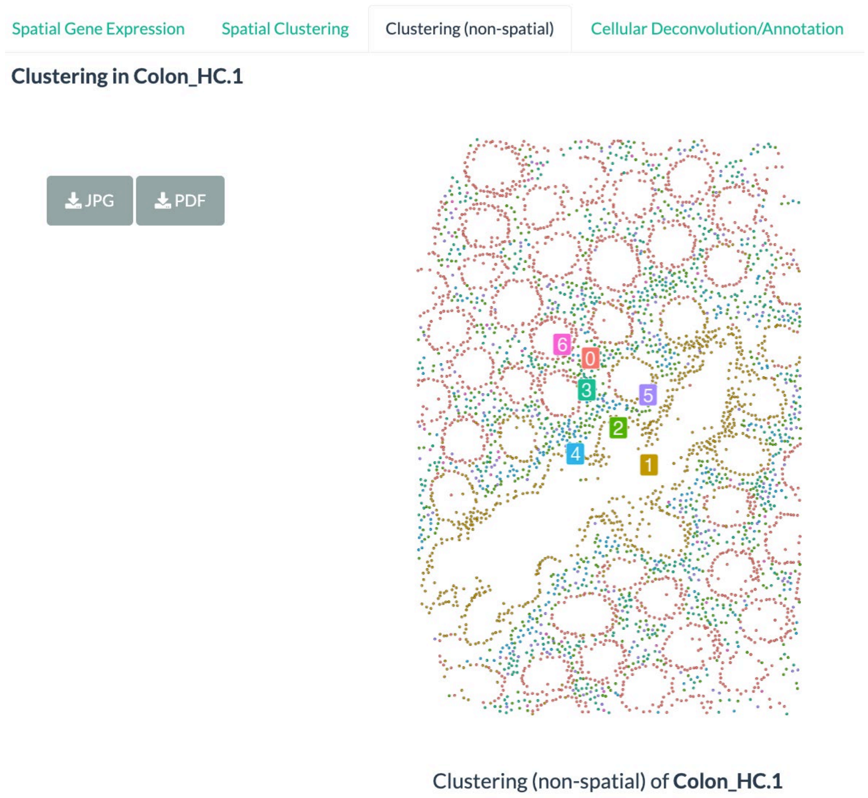

Fig. S11. Perform non-spatial clustering in SOAR's Data Browser. Clustering based on expression alone on the colon sample shows clusters that correlate with tissue structure and agreement with spatial clustering.

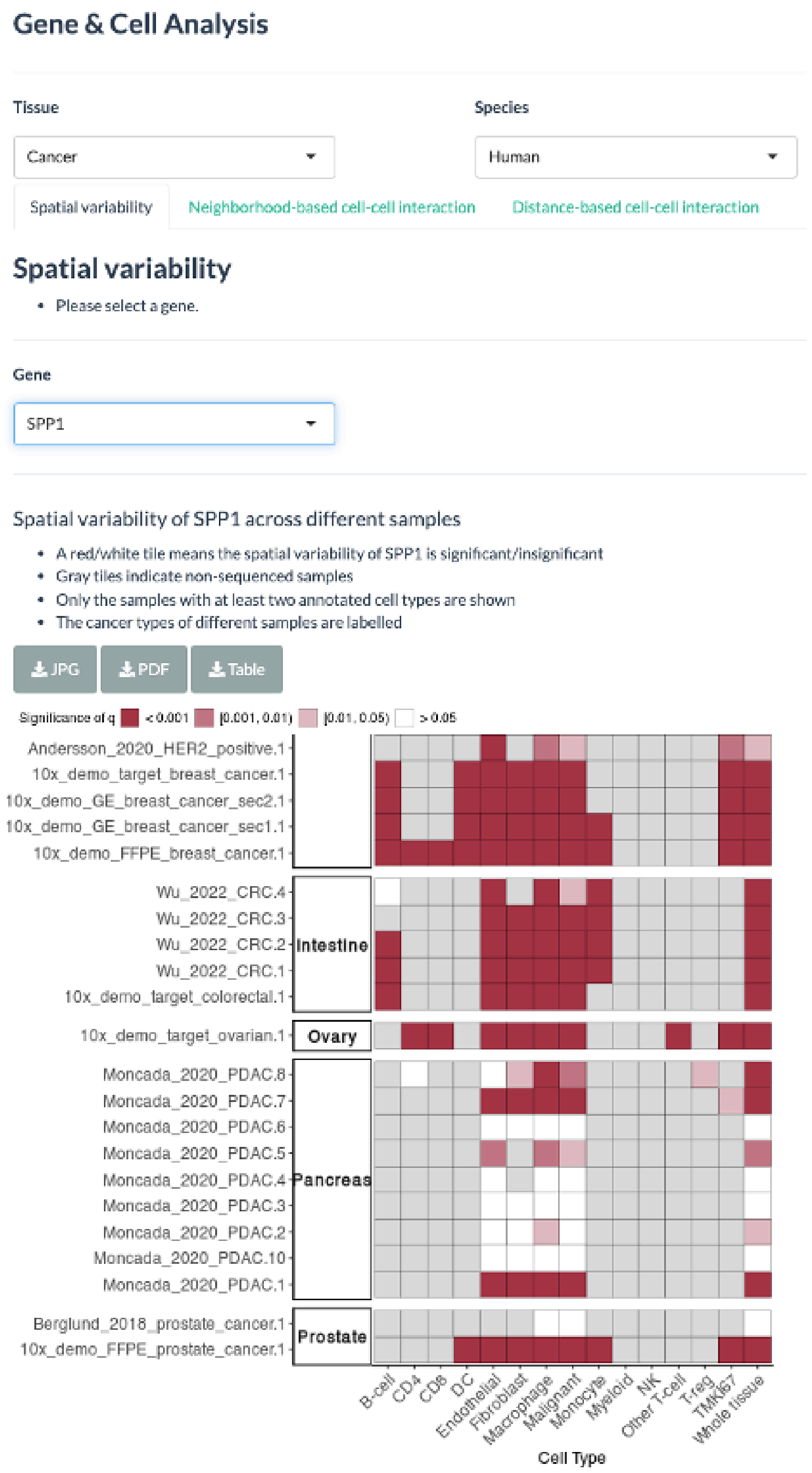

Fig. S12. Spatial variability function in SOAR's Gene & Cell Analysis module. Spatial variability allows for quantifiable mega-analysis of whether a gene is spatially variable at the cell

type and tissue level across samples of the same tissue and species. Note that this figure is for demonstration purposes only and the heatmap in this screenshot is cropped.

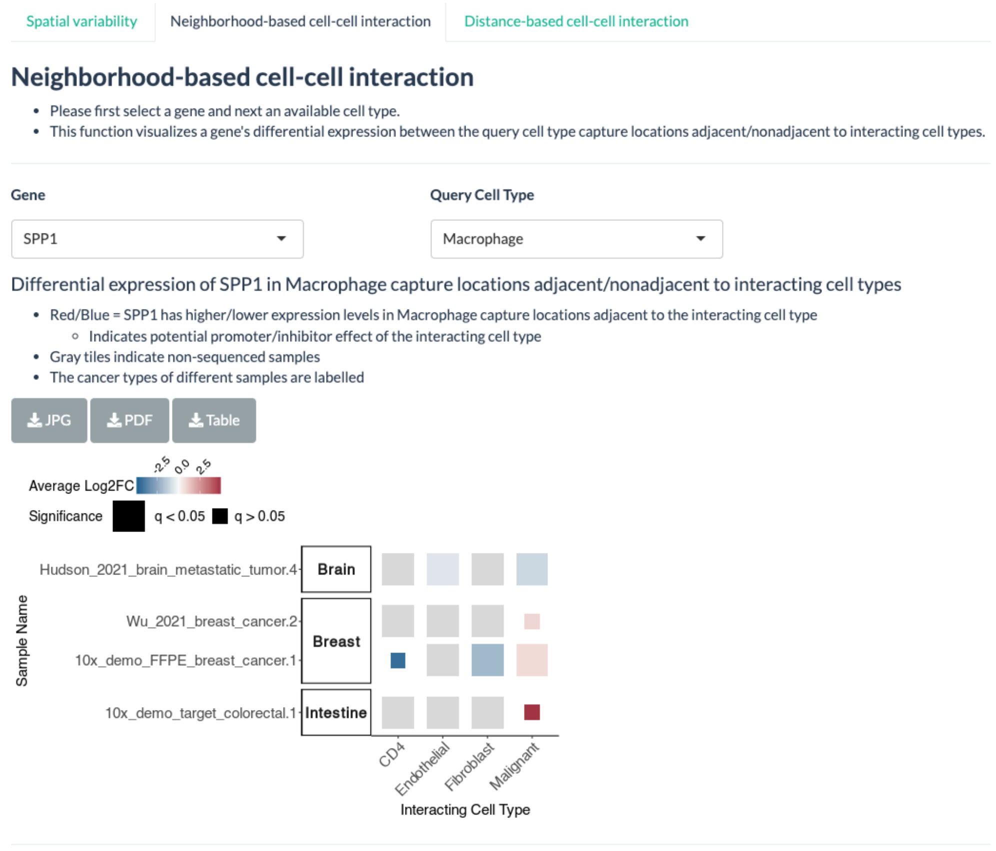

Fig. S13. Neighborhood-based cell-cell interaction function in SOAR's Gene & Cell Analysis module. Neighborhood-based cell-cell interaction evaluates whether a gene has higher expression in a cell type of interest when it is nearby another cell type.

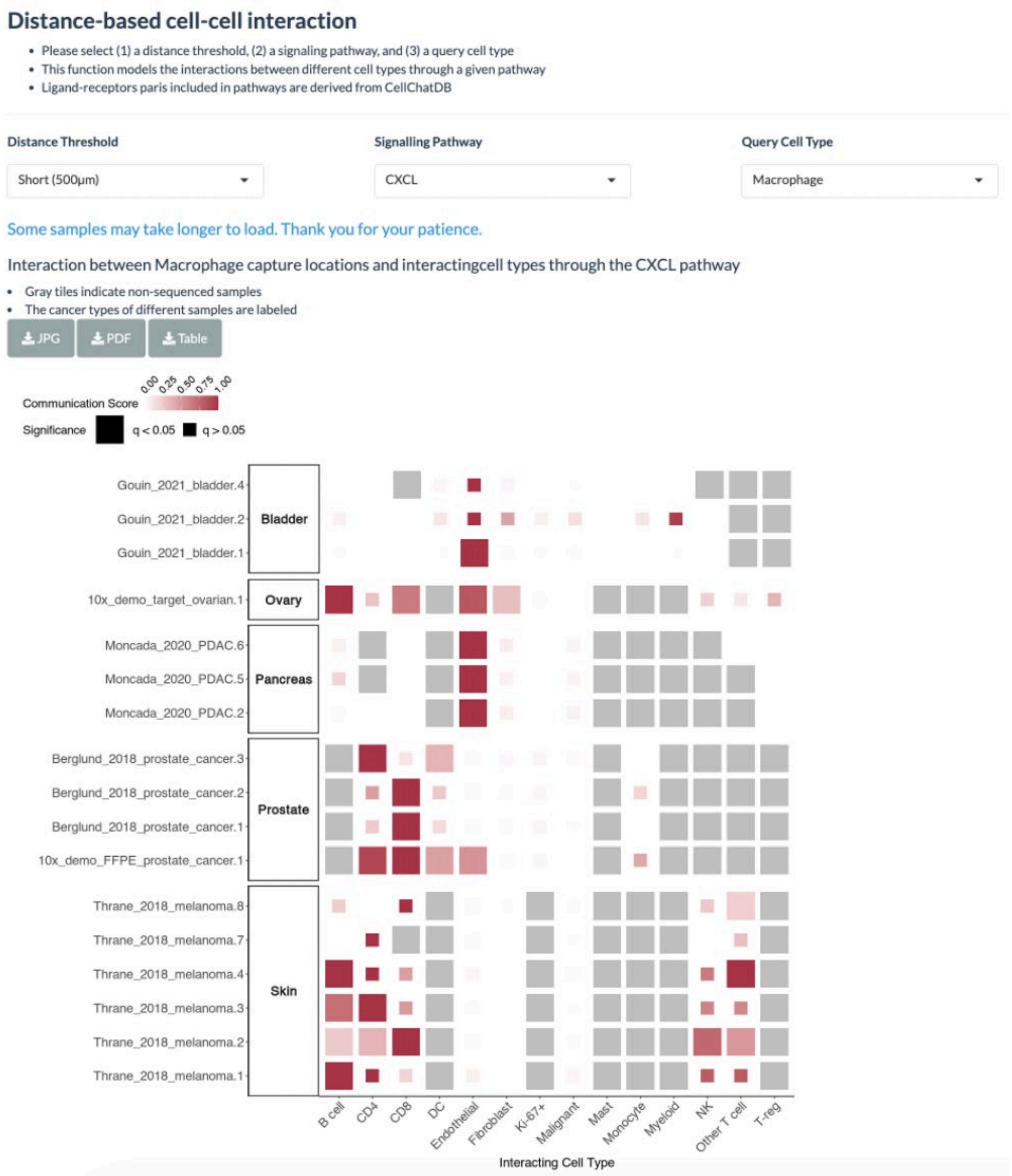

Fig. S14. Distance-based cell-cell interaction function in SOAR's Gene & Cell Analysis module. Spatial distance is further leveraged in distance-based cell-cell interaction as expression of a pathway in a cell type is modeled with various distance thresholds to another cell type.

Pathological Sample Browser

Search:

Dataset	Sample	Technology	Organ	Tissue	Condition	Cell Type	Direction	Compound	CMAP Instance	Enrichment Score
["10x_demo_GE_breast_canc	All	All	All	All	All	["Ma	All	All	All	All
10x_demo_GE_breast_cancer_sec1	1	Visium	Breast	Breast Cancer	Malignant	Malignant	Positive	everolimus	CPC014_HT29_6H:BRD-K13514097-001-01-2:10	0.413
10x_demo_GE_breast_cancer_sec1	1	Visium	Breast	Breast Cancer	Malignant	Malignant	Negative	BIX-02189	ERG011_VCAP_6H:BRD-K73368362-001-03-4:2	-0.365
10x_demo_GE_breast_cancer_sec1	1	Visium	Breast	Breast Cancer	Malignant	Malignant	Positive	BRD-A05831822	CPC008_HT29_6H:BRD-A05831822-001-01-4:10	0.389
10x_demo_GE_breast_cancer_sec1	1	Visium	Breast	Breast Cancer	Malignant	Malignant	Positive	BRD-A36275421	CPC006_NCIH508_6H:BRD-A36275421-001-11-1:10	0.39
10x_demo_GE_breast_cancer_sec1	1	Visium	Breast	Breast Cancer	Malignant	Malignant	Positive	BRD-K02952507	DOS022_VCAP_6H:BRD-K02952507-004-02-3:5.02	0.399
10x_demo_GE_breast_cancer_sec1	1	Visium	Breast	Breast Cancer	Malignant	Malignant	Negative	BRD-K03376048	PAC018_U2OS_6H:BRD-K03376048-001-01-8:9.99171	-0.418
10x_demo_GE_breast_cancer_sec1	1	Visium	Breast	Breast Cancer	Malignant	Malignant	Positive	BRD-K03568952	DOS025_VCAP_6H:BRD-K03568952-001-01-4:5.04	0.386
10x_demo_GE_breast_cancer_sec1	1	Visium	Breast	Breast Cancer	Malignant	Malignant	Positive	BRD-K05402890	PAC069_U2OS_6H:BRD-K05402890-001-08-4:20	0.455
10x_demo_GE_breast_cancer_sec1	1	Visium	Breast	Breast Cancer	Malignant	Malignant	Negative	BRD-K06716139	DOS022_VCAP_6H:BRD-K06716139-001-01-2:5.05	-0.365
10x_demo_GE_breast_cancer_sec1	1	Visium	Breast	Breast Cancer	Malignant	Malignant	Negative	BRD-K07303502	HOG003_MCF7_6H:BRD-K07303502-001-02-5:0.0412	-0.391

Showing 1 to 10 of 100 entries (filtered from 443,800 total entries)

Previous 1 2 3 4 5 ... 10 Next

*To download the full top/down 5,000 perturbation results table for exploration, please click link

Fig. S15. The Pathological Sample Browser in SOAR's Drug Discovery module. Only samples with pathological conditions are listed in the Pathological Sample Browser. Each row represents a CMap perturbation instance on a certain cell type of a pathological sample.

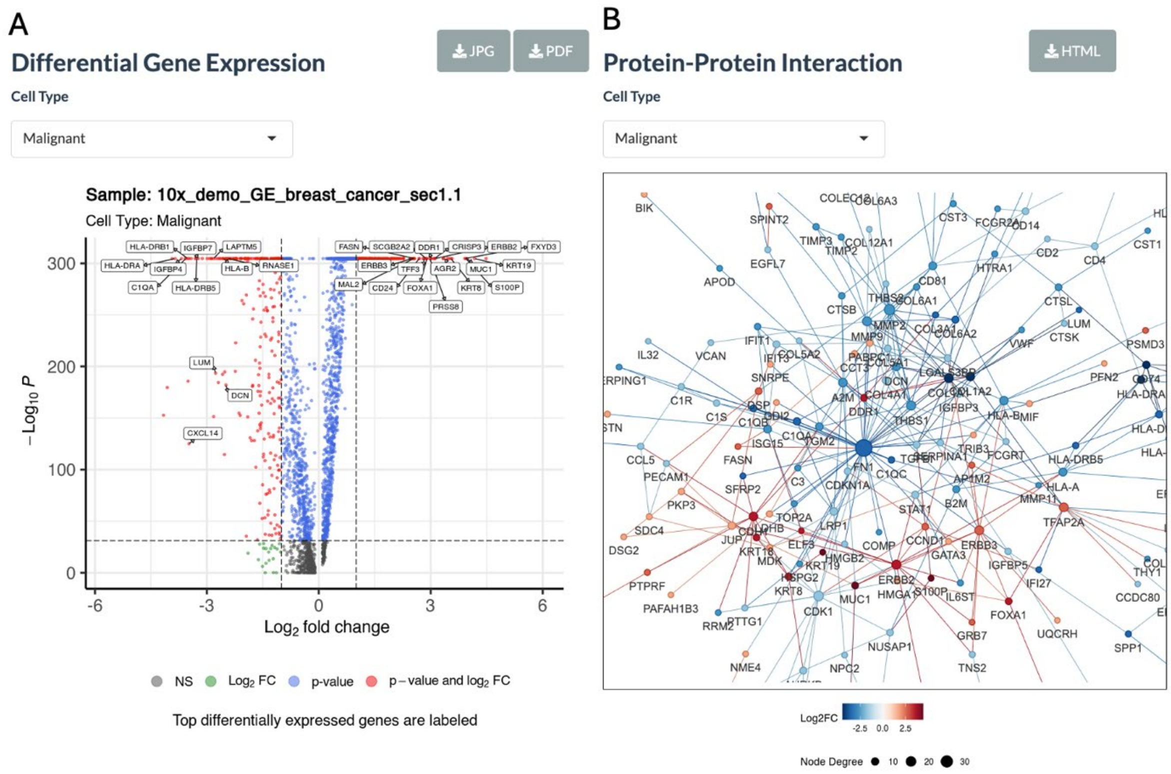

Fig. S16. Differential gene expression and protein-protein interaction functions in SOAR's Drug Discovery module. (A) Top and bottom spatially variable and differentially expressed genes (DEGs) are shown for each cell type of a pathological sample. **(B)** The network shows potential protein-protein interactions among spatially variable DEGs of a cell type.

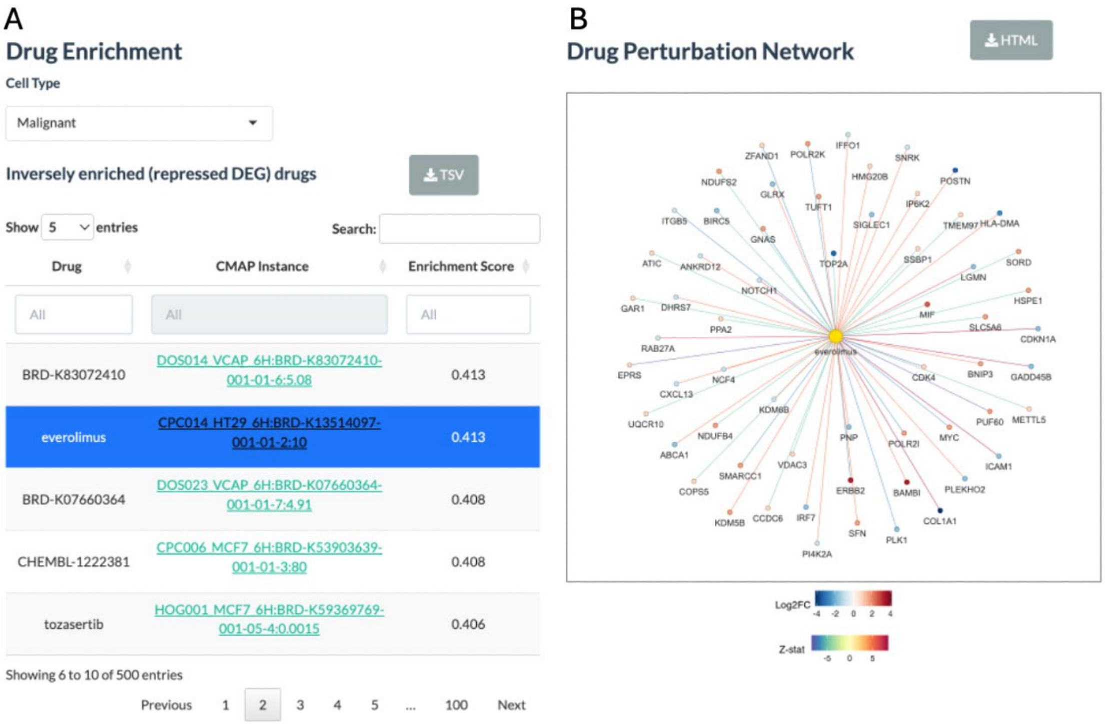

Fig. S17. Drug enrichment and drug perturbation network functions in SOAR's Drug Discovery module. (A) The lists of top and bottom 500 enriched allow users to identify compounds with strong repression or promotion effects on the DEGs of a cell type. **(B)** The network shows the direction of perturbation a compound has on the top and bottom 30 affected DEGs.

Supplementary Table Captions

Table S1 is in the attached Table S1.xlsx.

Table S1. Comparison of SOAR and other spatial transcriptomics resources. SOAR hosts the largest number of spatial transcriptomics samples with spatial coordinates data and provides the widest range of analysis capabilities. ALS, amyotrophic lateral sclerosis; CCI, cell-cell interaction; SV, spatial variability.

Table S2 is in the attached Table S2.xlsx.

Table S2. Detailed information of the datasets in SOAR. The dataset IDs are used in SOAR's "Data Browser" module. The table lists each dataset's spatial transcriptomics technology, species, and tissue type. For datasets associated with published papers and preprints, the corresponding PubMed IDs are noted, whereas, for online data collections, links to their sources are included in the table.
